# Stacked ensembles on basis of parentage information can predict hybrid performance with an accuracy comparable to marker-based GBLUP

**DOI:** 10.3389/fpls.2023.1178902

**Published:** 2023-07-21

**Authors:** Philipp Georg Heilmann, Matthias Frisch, Amine Abbadi, Tobias Kox, Eva Herzog

**Affiliations:** ^1^ Institute of Agronomy and Plant Breeding II, Justus Liebig University, Gießen, Germany; ^2^ NPZ Innovation GmbH, Holtsee, Germany

**Keywords:** machine learning, stacked ensembles, gradient boosting, genomic prediction, general combining ability, specific combining ability, hybrid breeding, hybrid prediction

## Abstract

Testcross factorials in newly established hybrid breeding programs are often highly unbalanced, incomplete, and characterized by predominance of special combining ability (SCA) over general combining ability (GCA). This results in a low efficiency of GCA-based selection. Machine learning algorithms might improve prediction of hybrid performance in such testcross factorials, as they have been successfully applied to find complex underlying patterns in sparse data. Our objective was to compare the prediction accuracy of machine learning algorithms to that of GCA-based prediction and genomic best linear unbiased prediction (GBLUP) in six unbalanced incomplete factorials from hybrid breeding programs of rapeseed, wheat, and corn. We investigated a range of machine learning algorithms with three different types of predictor variables: (a) information on parentage of hybrids, (b) in addition hybrid performance of crosses of the parental lines with other crossing partners, and (c) genotypic marker data. In two highly incomplete and unbalanced factorials from rapeseed, in which the SCA variance contributed considerably to the genetic variance, stacked ensembles of gradient boosting machines based on parentage information outperformed GCA prediction. The stacked ensembles increased prediction accuracy from 0.39 to 0.45, and from 0.48 to 0.54 compared to GCA prediction. The prediction accuracy reached by stacked ensembles without marker data reached values comparable to those of GBLUP that requires marker data. We conclude that hybrid prediction with stacked ensembles of gradient boosting machines based on parentage information is a promising approach that is worth further investigations with other data sets in which SCA variance is high.

## 1 Introduction

Hybrid breeding programs have been a decade-long success story in corn, but are also increasingly implemented in crops that have previously been commercialized as homozygous line varieties, such as wheat (Schulthess et al., 2017), barley (Philipp et al., 2016) or rapeseed (Stahl et al., 2017). By implementing hybrid breeding, breeders hope to improve performance, resilience and yield stability of their varieties. For maximizing heterosis and hybrid performance, the hybrid breeding material is usually arranged in so-called heterotic groups of individuals with similar combining ability and heterotic response when crossed to individuals from genetically distinct germplasm groups (Melchinger and Gumber, 1998). Two heterotic groups used in a specific hybrid breeding program are referred to as a heterotic pattern. Breeding progress and establishment of novel heterotic patterns is based on constant selection for hybrid performance, heterosis and combining ability in test crosses between the parent groups. In most breeding programs, the number of potential hybrid combinations of the parental lines from the heterotic groups exceeds the number of hybrids that can be evaluated in field trials by far.

By estimating the general combining ability (GCA, Hallauer et al., 2010) of the parental components, the performance of the resulting hybrids can be predicted using the sum of both parental GCA values. GCA estimates can be obtained by testing only a part of all possible crosses of parental lines from two different genetic groups in the field. If heterotic patterns have been established, candidates for hybrid parents can be very efficiently identified with only one or a few testers from the opposite heterotic group due to the high accuracy and predominance of GCA variance over special combining ability (SCA) variance (Melchinger and Gumber, 1998). The GCA prediction approach is simple, yet in many breeding programs surprisingly precise. For decades, it has formed the backbone of successful hybrid breeding programs.

However, newly established hybrid breeding programs usually cannot rely on established heterotic patterns. These hybrid programs are often characterized by a predominance of SCA variance over GCA variance, which complicates GCA-based testing strategies. Due to the high costs of evaluating large numbers of potential hybrid combinations in the field, genetic bottlenecks in one or both parent germplasm groups, and unsuccessful crosses without viable offspring, testcross factorials in these hybrid programs are often highly unbalanced and consist only of a small fraction of all possible hybrid combinations between the parent groups. As a consequence, new prediction methods that enhance the accuracy of hybrid prediction in sparse unbalanced factorials with high relevance of SCA are continually sought after to increase the efficiency of selection in newly established hybrid breeding programs.

Genomic prediction models for hybrid performance are able to incorporate information of genome-wide marker data in addition to phenotypic estimates collected in the field. These genomic prediction models have been successfully used to predict the testcross performance of untested parental lines (Albrecht et al., 2011; Hofheinz et al., 2012). For parent groups with a high ratio of SCA over GCA variance, as frequently observed in newly established hybrid breeding programs, modifications of the genome-wide BLUP (GBLUP) model incorporating both GCA and SCA components have been shown to increase prediction accuracy over models considering additive GCA effects only (Technow et al., 2012; Technow et al., 2014).

The term machine learning (ML) summarizes a large number of comparatively new prediction methods in statistics, mathematics, and computer science (Domingos, 2012). These methods have gained a lot of popularity due to their proven ability to solve problems in many different fields of research more effectively than classical approaches (Butler et al., 2018; Abbas et al., 2019; Dargan et al., 2020), but have not yet been widely implemented in hybrid breeding programs. A common feature of ML algorithms is that they are able to model non-linear interactions, and thus find complex underlying patterns within data better than other algorithms (Bishop, 2006; Hastie et al., 2009). Each algorithm has a wide variety of parameters that have to be manually defined by the user, so-called hyperparameters. Thus, an important part of the application of ML is the search for the optimal hyperparameters, which is generally known as hyperparameter optimization (Probst et al., 2019). This process requires knowledge on ML and, depending on the task and data set, a lot of computational resources.

Among the most popular ML algorithms are decision-tree based methods. Most commonly used are gradient boosting (GB, Friedman, 2001), which consists of several decision trees trained after another, extreme gradient boosting (XGB, Chen and Guestrin, 2016) which is a computationally more efficient version of GB and specialized in handling sparse data, and Random Forests (RF, Breiman, 2001), where multiple decision trees are trained in parallel. There is also the field of deep learning centered around the application of artificial neural networks (ANN, Goodfellow et al., 2016) that gained major popularity in the recent years. A classic type of ML algorithm are support vector machines (SVM, Cortes and Vapnik, 1995), which were first introduced as a classification algorithm but have later been adapted to regression tasks (Hastie et al., 2009). Reproducing kernel hilbert spaces (RKHS, Perez and de los Campos, 2014) are similar to SVM and are additionally already quite common in plant breeding. A very simple and fast algorithm focused on filling out sparse matrices is matrix factorization (MF, Koren et al., 2009), most commonly used in recommender systems. Stacked ensembles (SE, Breiman, 1996; Van Der Laan et al., 2007) utilize the output of already existing models to train a new model on top. This model combines aspects of all models it incorporates.

Recent studies have started to investigate the potential of ML for tasks related to plant breeding. ML has been used for handling genotype-by-environment interactions in multi-environmental trials (Montesinos-López et al., 2018b; Gillberg et al., 2019; Washburn et al., 2021; Westhues et al., 2021), the identification of the optimal set of markers used for prediction (Li et al., 2018a; Gabur et al., 2022), phenomic prediction and image classification (Mohanty et al., 2016; Nagasubramanian et al., 2018; Cuevas et al., 2019; Nagasubramanian et al., 2019) as well as genomic prediction (Ma et al., 2018; Azodi et al., 2019; Banerjee et al., 2020; Montesinos-López et al., 2021). The majority of recently published studies rely on genomic data as the basis of their predictions. Studies without genomic data usually incorporate other forms of complex data or prove the concept of a single specific algorithm without conducting a broad investigation of the potential of available algorithms (Montesinos-López et al., 2018a; Khaki and Wang, 2019; Khaki et al., 2020). To our knowledge, a comparison of ML methods under the same conditions as GCA-based hybrid prediction with real-life data from ongoing breeding programs has not yet been investigated.

Our goal was to investigate the suitability of the ML algorithms GB, RF, XGB, ANN, MF, RKHS, SVM, and an SE based on GB machines (GB-SE) for prediction of hybrid yield in six unbalanced factorials of different structure and size from hybrid breeding programs of rapeseed, wheat, and corn. In particular, our objectives were (i) to compare the prediction accuracy of ML algorithms based on hybrid parentage and phenotypic field data to classical GCA-based prediction, (ii) to test if the best ML algorithm from objective (i) can compete with marker-based predictions from a GBLUP model incorporating GCA and SCA components, (iii) to investigate if ML algorithms based on genotypic data or a combination of genotypic data and parentage information can outperform a GBLUP model incorporating GCA and SCA components, (iv) and to develop a user-friendly standardized procedure for hyperparameter optimization that is applicable in a wide range of hybrid breeding programs.

## 2 Material and methods

### 2.1 Software

All analyses were conducted in R 4.0.3 (R Core Team, 2022). For analysis of field data, GCA and SCA effects, GBLUP and implementation of the ML algorithms, we used the R packages ‘lme4 1.1-31’ (Bates et al., 2015), ‘emmeans 1.7.3’ (Lenth, 2021), ‘sommer 4.2.0’ (Covarrubias-Pazaran, 2016; Covarrubias-Pazaran, 2018), ‘h2o 3.38.0.1’ (LeDell et al., 2020; Chen et al., 2022), ‘kernlab 0.9-31’ (Karatzoglou et al., 2004), ‘mlr 2.19.0’ (Bischl et al., 2016), ‘parallelMap 1.5.1’ (Bischl et al., 2020), ‘BGLR 1.1.0’ (Perez and de los Campos, 2014), and ‘recosystem 0.5’ (Qiu et al., 2021), which are available from the Comprehensive R Archive Network (CRAN). Additionally, we used the package ‘SelectionTools 21.3’ which is freely available under http://population-genetics.uni-giessen.de/software/. For which algorithms the specific packages were used is described in detail below. A working R code example for tuning GB models with random grid search and building the GB-SE is provided for data set Co1 as PDF in the **supplementary material**, and as an R script under https://github.com/PGHeilmann/Minimalist-ML-frontiers.

### 2.2 Experimental data sets

We investigated six experimental data sets of hybrid yield which comprised incomplete factorials of two unbalanced parent groups. Descriptive statistics for the investigated factorials are summarized in **Table 1**. A graphical overview of the crossing matrices and the realized hybrid combinations for the six factorials is given in **Figures S1**, **S2**.

**Table 1:** Descriptive statistics, variance components and proportion of SCA variance of the total variance τ for the six experimental data sets.

data set	Ra1	Ra2	Ra3	Wh1	Co1	Co2
No. of parents in group 1	381	756	516	120	123	50
No. of parents in group 2	14	24	29	15	86	41
Ratio group 1/group 2	27.2	31.5	17.8	8.0	1.4	1.2
No. of possible hybrids	5334	18144	14964	1800	10578	2050
No. of realized hybrids	746	1621	1081	1604	1254	550
Fraction of realized hybrids	14.0%	8.9%	7.2%	89.1%	11.9%	26.8%
Group 1: no. of crosses per parent						
Mean	2.0	2.1	2.1	13.4	10.2	11.0
Median	2.0	2.0	2.0	14.0	7.0	11.0
Range	1-6	2-5	1-6	3-15	2-55	3-26
Group 2: no. of crosses per parent						
Mean	53.3	67.5	37.3	106.9	14.6	13.4
Median	34.5	22.5	6.0	107.0	13.0	13.0
Range	2-140	2-247	1-222	91-117	1-99	2-42
Heterotic pools	No	No	No	No	Yes	Yes
$r\left(GCA_{1i}+GCA_{2j},\text{Hybrid yield}\right)$	0.67	0.82	0.92	0.76	0.91	0.94
$r\left(SCA_{ij},\text{Hybrid yield}\right)$	0.93	0.88	0.69	0.70	0.49	0.40
Variance components						
$\sigma_{{\text{GCA}}_{1}}^{2}$	0.516	5.50	12.95	0.048	43.21	73.05
$\sigma_{{\text{GCA}}_{2}}^{2}$	0.774	3.32	2.20	0.024	20.25	24.39
$\sigma_{\text{SCA}}^{2}$	2.663	9.35	5.90	0.051	17.47	15.78
$\tau =\sigma_{\text{SCA}}^{2}/\left(\sigma_{\text{GCA}1}^{2}+\sigma_{\text{GCA}2}^{2}+\sigma_{\text{SCA}}^{2}\right)$	0.67	0.51	0.28	0.44	0.22	0.14

The phenotypic and genotypic data of rapeseed factorials Ra1 - Ra3 was provided by Norddeutsche Pflanzenzucht Hans-Georg Lembke KG. Factorial Ra1 consisted of 746 realized hybrids derived from two parent groups with 381 and 14 inbred lines. Factorial Ra2 consisted of 1621 realized hybrids derived from two parent groups with 756 and 24 inbred lines. Factorial Ra3 consisted of 1081 realized hybrids derived from two parent groups with 516 and 29 inbred lines. Phenotypic yield data was provided as adjusted entry means for each hybrid.

The phenotypic and genotypic data of wheat factorial Wh1 was published in Zhao et al. (2015) and Gowda et al. (2014). Factorial Wh1 consisted of 1604 realized hybrids derived from two parent groups with 120 and 15 inbred lines. Phenotypic yield data was provided as adjusted entry means for the hybrids in 11 environments. We calculated adjusted entry means for hybrid yield over environments with the mixed linear model $y_{ij}=\mu +g_{i}+e_{j}+\epsilon_{ij}$ , where μ is the population mean, $g_{i}$ is the fixed effect of the i -th hybrid genotype, $e_{j}$ the random effect of the j -th environment, and $\epsilon_{ij}$ is the residual error. We did not include a genotype-by-environment interaction term, as the published data consisted of environment-specific adjusted entry means of the hybrids, which already included the replications within environments. We used the R packages ‘lme4 1.1-31’ (Bates et al., 2015) and ‘emmeans 1.7.3’(Lenth, 2021) for fitting the model and calculating the adjusted entry means.

The phenotypic and genotypic data of corn factorial Co1 was published in Technow et al. (2014) and accessed through the R package ‘sommer 4.2.0’(Covarrubias-Pazaran, 2016). Factorial Co1 consisted of 1254 hybrids derived from two parent groups with 123 and 86 inbred lines. Phenotypic yield data was provided as adjusted entry means for each hybrid.

The phenotypic and genotypic data of corn factorial Co2 was published in Schrag et al. (2018). Factorial Co2 consisted of 550 hybrids derived from two parent groups with 50 and 41 inbred lines. Phenotypic yield data was provided as adjusted entry means for each hybrid.

### 2.3 Pre-processing of genotypic marker data

Genotypic marker data was only available for factorials Ra1, Wh1, Co1 and Co2. For all four factorials, genotypic data consisted of single nucleotide polymorphisms (SNPs). The original marker data consisted of 52157 (Ra1), 1280 (Wh1), 35478 (Co1), and 37392 (Co2) SNP markers, respectively. Markers were removed from a data set if expected heterozygosity was below 10%, or if more than 1% of entries were missing. The remaining missing data was imputed using the mean of the respective marker. ‘SelectionTools 21.3’ was used for filtering and ‘sommer 4.2.0’ for imputing the data. For all data sets, it was checked that genetic markers evenly covered the whole genome. After pre-processing, 10880 (Ra1), 1264 (Wh1), 26069 (Co1) and 33666 (Co2) SNP markers remained for further analysis.

### 2.4 Linear model for GCA and SCA effects

The GCA of the parents ${\text{GCA}}_{1i}$ and ${\text{GCA}}_{2j}$ , and the SCA of the hybrids ${\text{SCA}}_{ij}$ were predicted by BLUP with the mixed linear model


$$y_{ij}=\mu +{\text{GCA}}_{1i}+{\text{GCA}}_{2j}+{\text{SCA}}_{ij}$$

where $y_{ij}$ is the (adjusted) treatment mean of the hybrid of the i -th parent from parent group 1 and the j -th parent from parent group 2, μ is the population mean, ${\text{GCA}}_{1i}$ is the random GCA effect from the i -th parent from parent group 1, ${\text{GCA}}_{2i}$ is the random GCA effect from the j -th parent from parent group 2, ${\text{SCA}}_{ij}$ is the SCA effect. We also used this model for estimating the GCA and SCA variances. We used the R package ‘lme4 1.1-31’ (Bates et al., 2015) for fitting the model and predicting the GCA and SCA as well was the corresponding variance components.

To evaluate the relevance of GCA and SCA for hybrid yield in the single factorials, we calculated the sum ${\text{GCA}}_{1i}+{\text{GCA}}_{2j}$ for each realized hybrid, the Pearson correlations $r\left({\text{GCA}}_{1i}+{\text{GCA}}_{2j},\text{Hybrid yield}\right)$ and $r\left({\text{SCA}}_{ij},\text{Hybrid yield}\right)$ for all realized hybrids, and the proportion of the contribution of the SCA variance to the total genetic variance in the factorial $\tau =\sigma_{\text{SCA}}^{2}/\left(\sigma_{{\text{GCA}}_{1}}^{2}+\sigma_{{\text{GCA}}_{2}}^{2}+\sigma_{\text{SCA}}^{2}\right)$ (**Table 1**).

### 2.5 Mixed model for GBLUP

Using the adjusted entry means for the hybrids from field trial analysis as phenotypic inputs y , we fitted a GBLUP model including GCA and SCA effects (Technow et al., 2014):


$$y=1\beta_{0}+Z_{1}g_{1}+Z_{2}g_{2}+Z_{S}s+e$$

where $\beta_{0}$ is a fixed intercept, $Z_{1}$ and $Z_{2}$ are the incidence matrices for the parents from parent groups 1 and 2, $Z_{S}$ is the incidence matrix for the hybrids, $g_{1}$ and $g_{2}$ are vectors of random GCA effects from the parental lines from group 1 and 2, s is the vector of the random SCA effects for the hybrids, end e is the vector of residual errors. The genomic relationship matrices $G_{1}$ and $G_{2}$ for $g_{1}$ and $g_{2}$ were calculated as ${\text{G}}_{1}={\text{W}}_{1}{{\text{W}}^{\prime }}_{1}/\text{c}$ and ${\text{G}}_{2}={\text{W}}_{2}{{\text{W}}^{\prime }}_{2}/\text{c}$ , where $w_{uv}=x_{uv}+1-2p_{v}$ and $c=2\sum_{v}p_{v}\left(1-p_{v}\right)$ , where u is the index of the parent, v is the index of the marker, $x_{uv}$ the coding number of the genotype of parent u at marker locus v , i.e. -1 or 1, and $p_{v}$ the allele frequency of the 1 allele in the respective parent group (Endelman and Jannink, 2012). The genomic relationship matrix S for s was calculated as the Kronecker product $G_{1}⊗G_{2}$ in accordance to Stuber and Cockerham (1966). We used the R package ‘sommer 4.2.0’ 2 (Covarrubias-Pazaran, 2016; Covarrubias-Pazaran, 2018) for fitting the GBLUP model and predicting hybrid yields.

### 2.6 ML algorithms

#### 2.6.1 Input variables

For the ML algorithms, we investigated three different scenarios: prediction of hybrid yield without genotypic information, prediction of hybrid yield with genotypic marker data, and a combined set of variables. For prediction without genotypic information, we investigated two different sets of input variables. The parentage-based set of input variables consisted of the nominal parent factor levels, i.e. names or barcodes of the parent lines. For each hybrid, the only available information were the names of its parents. Thus, the original set of input variables consisted of only two variables, which for some algorithms were converted to binary variables via one-hot encoding.

For the second set of input variables, we again determined the two parents of each hybrid. The input variables consisted of the hybrid yields of each parent from the available crosses with all parents from the opposite parent group. Thus, this set of continuous input variables consisted of as many variables as the sum of the number of parents in the two parent groups. The hybrid yield in the response variable in a specific row of the data set was always deleted from the input variables in this row.

For prediction with genotypic marker data, we used the incidence matrix of the virtual hybrid genotypes for the pre-processed marker data coded with -1, 0, and 1 for homozygous for the first allele, heterozygous, and homozygous for the second allele as input variables.

For the combined set of variables, we merged the parentage-based set of input variables with the genotypic marker data and used all available information as input variables for the models.

#### 2.6.2 Investigated ML algorithms for different sets of input variables

The algorithms MF, SVM, GB, RF, ANN, and GB-SE were investigated with the parentage and the hybrid yields input variable sets. The algorithms XGB, XGB-SE, RKHS and SVM were investigated with genotypic marker data as input variables.

#### 2.6.3 Hyperparameters

A comprehensive overview over all the hyperparameter values considered for each ML algorithm is given in **Table S1**.

For GB, we tuned the number of decision trees (n_trees), the maximum depth of the trees (max_depth), the minimum number of observations per split (min_rows), the sample rate of observations per tree (sample_rate), and the number of bins for categorical variables (nbins_cats). We manually set the learning rate (learn_rate) to a constant value of 0.1 and used the default settings for all other hyperparameters.

For XGB, we essentially tuned the same hyperparameters as for GB, but used a fixed number of trees and tuned the learning rate. When XGB was only used with genotypic marker data, the hyperparameter nbins cats was removed. In this case the pruning parameter γ (gamma) was added instead. To handle the high dimensionality of the markers, random column sampling per tree (col sample rate by tree) was introduced.

Early stopping was used to reduce the computational time required.

For RF, we considered the same hyperparameters as for GB, with the exception that a learning rate does not exist for RF.

For ANN, we used the classical multilayer perceptron architecture for ANN (Goodfellow et al., 2016). We tuned the number of hidden layers and nodes within the hidden layers (hidden), the learning rate (rate), the number of iterations (epochs) and added input dropout (input_dropout) to some models. We used the rectified linear unit activation function for all models. The categorical variables of the parentage variable set were by default converted to binary variables by one-hot encoding.

For the GB-SE, we used ridge regression as the super learner and a certain optimal number of GB models selected by the grid search procedure and criteria described below as inputs.

The algorithms GB, XGB, RF, ANN and GB-SE were implemented using ‘h2o 3.38.0.1’ for R (LeDell et al., 2020; Chen et al., 2022).

For SVM, we tuned the hyperparameters ϵ (epsilon) and C (C). We considered three different kernels: linear, polynomial and radial. For the polynomial kernel, the degree of the polynomial was tuned. SVM was implemented using the R package ‘kernlab 0.9-31’ (Karatzoglou et al., 2004) with packages ‘mlr 2.19.0’ (Bischl et al., 2016) and ‘parallelMap 1.5.1’ (Bischl et al., 2020) for tuning and parallelization.

For RKHS, we used three gaussian kernels with bandwidth parameters 0.1, 0.5 and 2.5, respectively. We used the default function settings for all other parameters. RKHS was calculated using the package ‘BGLR 1.1.0’ [54]. RKHS did not require hyperparameter tuning.

For MF, we tuned the number of latent variables (dim), and used l1 regularization for some models during training, (costq_l1, costp_l1). The learning rate (lrate) was manually set to a constant value of 0.05 and the number of iterations (niters) to 500.

Categorical variables were encoded as numbers before being passed to the algorithm. MF was implemented using the package ‘recosystem 0.5’ (Qiu et al., 2021).

### 2.7 Random grid search

In order to determine the optimal set of hyperparameters for a given data set and ML algorithm, we performed a random grid search over a large hyperparameter space (**Table S1**) for every ML algorithm except RKHS, which did not require tuning. As a stopping criterion, we set a maximum number of 50 models. Thus, for each ML algorithm that required tuning, 50 models were trained, each with a randomly chosen set of hyperparameters from the hyperparameter space. The performance of these 50 models within the gridsearch was evaluated with a 10-fold cross validation for the respective training set. We used the mean squared error (MSE) to evaluate the 50 hyperparameter combinations, since this metric was available for all algorithm implementations we used. The hyperparameter combination with the lowest MSE was considered optimal and used to predict the hybrid yields of the test set.

We used the 50 models created in the random grid search for GB to build the GB-SE. To choose the optimal number of models to include in the GB-SE, we used an iterative process where the best 5,10,…,50 models were included in the GB-SE, and thenevaluated with the Pearson correlation $r\left(y_{ij},{\hat{y}}_{ij}\right)$ between the observed and predicted yield in a 10-fold cross validation. The optimum number of models to include in the final GB-SE was chosen according to the highest value of $r\left(y_{ij},{\hat{y}}_{ij}\right)$ .

### 2.8 Cross validation, pre-processing of training sets and test sets, and prediction of test set hybrids

All investigated prediction models and ML algorithms were tested in a cross validation procedure in order to evaluate the generalizability and stability of the predictions, and to evaluate the consistency of the applied grid search procedure for model selection. For cross validation, the respective factorial was randomly split into a training set consisting of 90% of the available hybrids, and a test set consisting of the remaining 10% of hybrids. This random split was repeated 100 times for each factorial. We removed all hybrids from the test set for which only one or none of the parents were represented in the training set as GCA estimation requires both parents to be available in the training set. If hybrid yields were used as input variables, all hybrid yields from the test set hybrids were removed from the training set.

After pre-processing, each of the investigated prediction models and ML algorithms was trained on the 100 training sets. The resulting models were used to predict the hybrid yields of the test set hybrids. For GCA prediction, the yield of the test set hybrids was predicted as ${\hat{y}}_{ij}=\mu +GCA_{1i}+GCA_{2j}$ . For GBLUP and the ML algorithms, the yield of the test set hybrids ${\hat{y}}_{ij}$ was predicted with the respective prediction routines implemented in the R packages.

### 2.9 Evaluation criteria for model performance and comparisons across algorithms

To evaluate and compare model performance across prediction models and ML algorithms, we calculated the Pearson correlation $r\left(y_{ij},{\hat{y}}_{ij}\right)$ between the observed and predicted yield of the test set hybrids. This correlation is referred to as “prediction accuracy”. For each method and factorial, we also compared the 20 best predicted hybrids to the 20 best observed hybrids and determined the percentage of overlap, since accurate identification of the best hybrids is more relevant to breeders than accurate predictions of low performing hybrids.

## 3 Results

### 3.1 Predictions based on parentage information and hybrid yields

**Figure 1:**
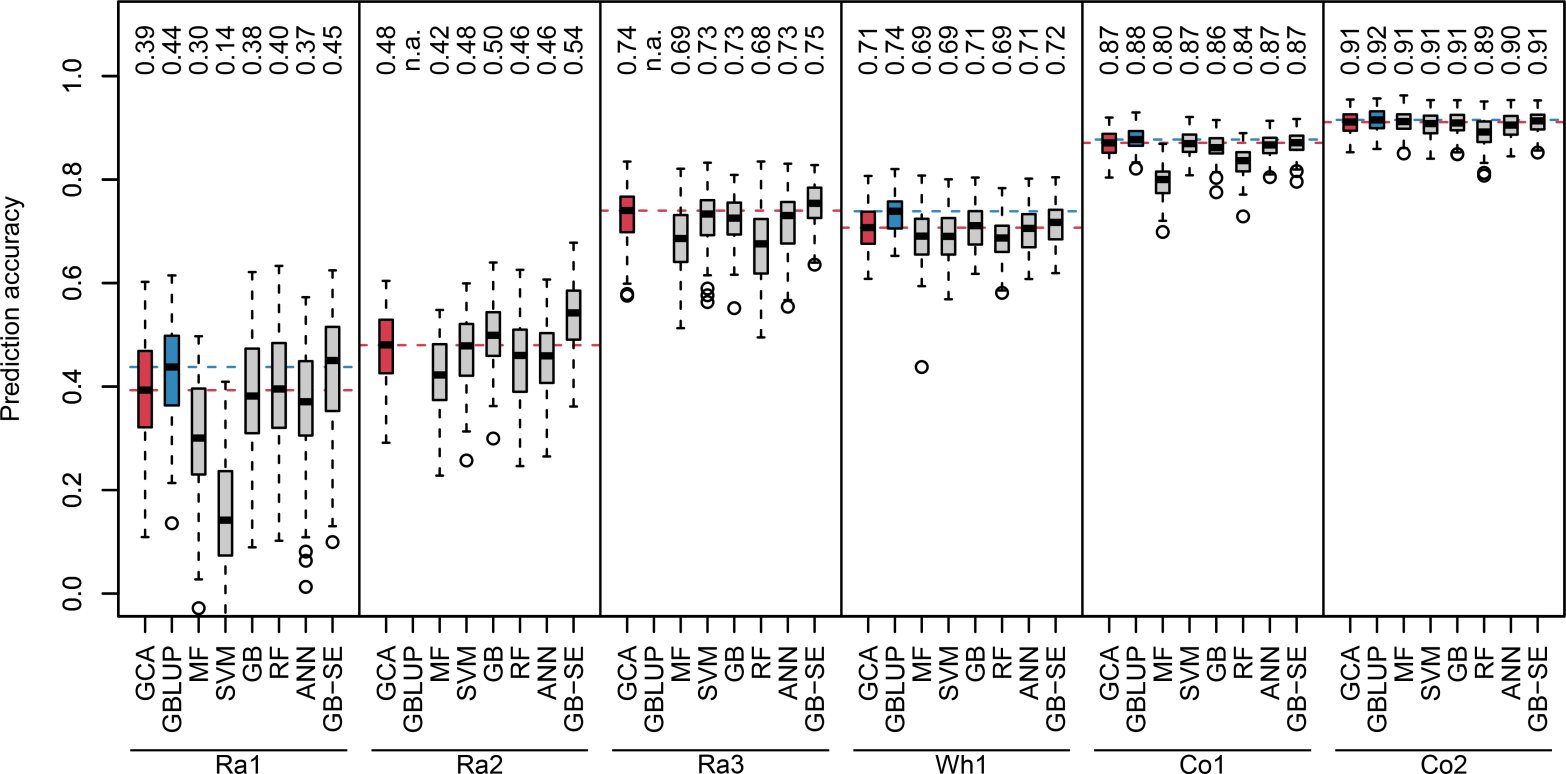
Boxplots of observed prediction accuracies in 100 cross validation splits for six different data sets (Ra1, Ra2, Ra3, Wh1, Co1, Co2) using eight different algorithms (GCA, GBLUP, MF, SVM, GB, RF, ANN, GB-SE). Median prediction accuracy is displayed above each boxplot. Median prediction accuracy of the baseline algorithms are represented by a dotted red (GCA) and blue (GBLUP) line. n.a., not available.

We investigated two different sets of input variables without genotypic information: parentage information, and yields of all other realized crosses of the parents of a specific hybrid. For algorithms using the parentage information as input variables, we observed a wide range of median prediction accuracies for the different crops and factorials (**Figure 1**, red and grey boxplots). The lowest overall prediction accuracieswere observed for the factorials Ra1 and Ra2, the median prediction accuracies ranging between 0.14 and 0.45, and between 0.42 and 0.54, respectively. The factorials Ra3 and Wh1 resulted in intermediate median prediction accuracies between 0.68 and 0.75,and 0.69 and 0.72, respectively. The factorials Co1 and Co2 resulted in the highest median prediction accuracies, ranging between 0.80 and 0.87, and 0.89 and 0.91, respectively.

GB was the best single ML algorithm for most investigated factorials with the exception of Ra1 and Co1, where RF and SVM performed better. MF resulted in the lowest median prediction accuracies for all factorials except Ra1. None of the investigated single ML algorithms resulted in higher median prediction accuracies than classical GCA prediction (**Figure 1**, red vs. grey boxplots). The GB-SE increased median prediction accuracies in the factorials with the overall lowest prediction accuracies Ra1 and Ra2 from 0.39 to 0.45, and from 0.48 to 0.54, respectively. For all other factorials, the median prediction accuracy of the GB-SE was equivalent to or only marginally better than GCA prediction.

Algorithms using hybrid yields as input variables increased computation time in comparison to algorithms based on parentage, but resulted in slightly lower median prediction accuracies (**Figure S3**). The algorithm ANN did not converge for all factorials with this set of input variables.

### 3.2 Comparison of GCA prediction, GB-SE and GBLUP

GBLUP was only investigated for the factorials Ra1, Wh1, Co1 and Co2 with available marker data (**Figure 1**, blue boxplots). Median prediction accuracies of GBLUP were equivalent to GCA prediction and the GB-SE in the factorials Co1 and Co2. In factorial Wh1, GBLUP slightly improved median prediction accuracy from 0.71 with GCA and 0.72 with the GB-SE to 0.74. In factorial Ra1, GBLUP increased median prediction accuracy from 0.39 with GCA to 0.44, and resulted in an equivalent prediction accuracy as the GB-SE with 0.45. Neither ML algorithms nor GBLUP did reduce the variation of prediction accuracies across cross validation splits in comparison to GCA prediction. Variation was generally low across cross validation splits for all investigated algorithms in factorials Wh1,Co1 and Co2, and largest in factorial Ra1 (**Figure 1**). For data sets Ra1, Ra2, Ra3, and Co1, GB-SE showed the highest percentage of overlap between the 20 best predicted and observed hybrids (**Figure S9**). For Wh1 and Co2, GBLUP showed the highest percentage of overlap. However, this percental overlap between the 20 best predicted and observed hybrids was generally similar for most algorithms with the exception of SVM and MF, which also performed poorly overall.

### 3.3 Effects of structure and composition of experimental data sets

The experimental data sets varied considerably in size, unbalancedness of the parent groups, percentage of realized hybrid combinations and relevance of GCA and SCA for hybrid yield (**Table 1**). As general trends, we observed that median prediction accuracies were high if the factorials relied on a heterotic pattern (Co1 and Co2), if percentage of realized hybrid combinations was high, i.e. if the factorial was almost complete (Wh1), if parent groups were more balanced, and if parentlines were represented in many hybrid crosses (Wh1, Co1 and Co2, compare **Table 1** with **Figure 1**). Importantly, GBLUP and the GB-SE increased prediction accuracy considerably in comparison to GCA prediction for the factorials with the highest proportion of SCA variance in the total variance (Ra1 and Ra2). For these factorials, the correlation of SCA with hybrid yield was higher than the correlation of GCA with hybrid yield (**Table 1**), and overall prediction accuracy was low (**Figure 1**). For all other factorials, there was no improvement in comparison to classical GCA prediction.

### 3.4 Performance of ML algorithms with genotypic information

**Figure 2:**
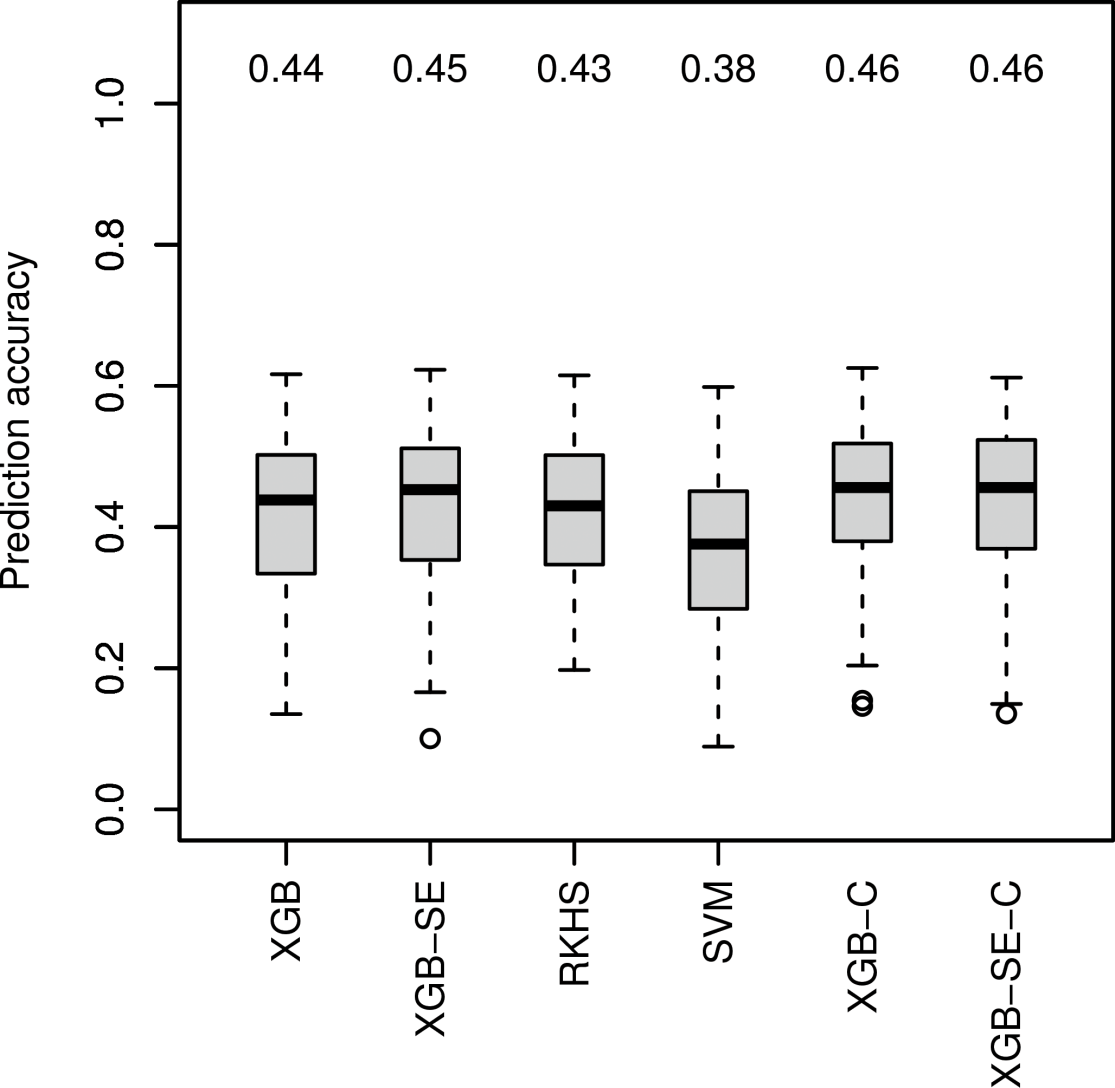
Comparison of observed prediction accuracies in 100 cross validation splits of marker-based ML algorithms for data set Ra1. Median prediction accuracy displayed above each boxplot. ’C’ indicating models were trained on the combined variable set.

For data set Ra1 with the highest improvement of median prediction accuracy with ML algorithm GB-SE and GBLUP (**Figure 1**), we investigated four additional algorithms specifically tuned for marker data: XGB, RKHS, SVM and an XGB-SE (**Figure 2**), and a combined set of input variables including marker data and parentage information for the best ML algorithms, XGB-C and XGB-SE-C. With marker data only, XGB and the XGB-SE resulted in the highest prediction accuracies of 0.44 and 0.45, but did not improve prediction accuracy compared to the GB-SE based on parentage information and GBLUP. For the algorithms based on a combination of marker data and parentage information, XGB-C and XGB-SE-C, prediction accuracy was increased slightly to 0.46. The ensemble did not show any improvement over the single algorithm anymore. In spite of the marginal increase in prediction accuracy, the ML methods based on marker data or the combination of marker data and parentage information did not result in a higher percental overlap of the predicted and observed 20 best hybrids (**Figure S9**).

### 3.5 Tuning and importance of different hyperparameters for ML algorithms

**Figure 3:**
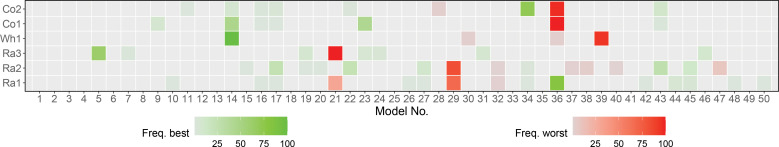
Selection of 50 models used in 100 cross validation splits for each data set using GB. Each model is corresponding to a unique random hyperparameter combination. Heatmap with green and red indicating how often a model was found to be the best or worst model over all iterations.

The random grid search approach we used for model tuning yielded overall consistent results for the same data set across cross validation splits, as reflected in the heatmap in **Figure 3** and **Table S3**. For most factorials, the same modelswere identified as the best and worst model in the majority of the cross validation splits. Where several models were identified as best, they were often similar for the most important hyperparameters. As a stopping criterion for random grid search, we set a maximum number of 50 models, which in the case of GB also formed the basis for building the GB-SE. With the exception of factorials Co1 and Co2, which in most cases used less than 25 models, the GB-SE was for most factorials and cross validation splits build from 30 or more models (**Figure S10**). The factorials Ra2 and Ra3 even required 40-50 models in the majority of cross validation splits.

For evaluating the importance of different hyperparameters, we calculated correlations between hyperparameter levels and MSE (**Table 2**) and created ridge line plots for each hyperparameter level and the scaled MSE (**Figures S4**-**S8**). We also compared the best models for the different data sets from the random grid search approach (**Table S2**). For conciseness, we only looked at the hyperparameters for the most successful single ML algorithm GB, which also formed the basis for building the GB-SE. The hyperparameters with the greatest effect on the MSE were the number of bins for partitioning the data before determining the tree’s best split point, and the maximum depth of the decision trees. As general trends, we observed that choosing an intermediate number of bins, ranging between 25-50% of the number of parents in the data set, resulted in the lowest MSE (**Table S2**). With respect to the maximum depth of the decision trees, we observed that the factorials Ra1-3 required deeper trees than the factorials Wh1, Co1 and Co2.

**Table 2:** Correlations between numerical hyperparameter levels and MSE of the models in the grid search.

Experiment	nbins_cats	max_depth	ntrees	sample_rate	min_rows
Ra1	0.62	-0.01	0.41	0.00	-0.08
Ra2	0.70	0.01	0.39	0.03	-0.09
Ra3	-0.33	0.00	0.21	-0.25	-0.11
Wh1	-0.38	0.06	0.07	-0.25	-0.34
Co1	-0.09	-0.03	0.19	-0.16	0.00
Co2	-0.22	-0.11	0.24	0.00	-0.18

## 4 Discussion

The present study is a case study exploring the potential of ML algorithms for prediction of hybrid yield in small unbalanced factorials. In accordance with the “No Free Lunch” theorem, implying that there is no single optimal algorithm for all problems and data sets, we tested a variety of single ML algorithms (MF, SVM, RF, GB, ANN, XGB, RKHS), as well as stacked ensembles of gradient boosting machines (GB-SE and XGB-SE) with optimized hyperparameters in comparison to the well-established approaches GCA prediction and GBLUP. The experimental data sets consisted of six unbalanced factorials from four different self-pollinating and outcrossing crops, and varied considerably in structure and size (**Table 1**, **Figures S1** and **S2**). The parent groups were highly unbalanced with respect to the number of parent lines in the groups for some of the factorials, while the parent groups were almost balanced in size for other factorials, the ratios of group sizes ranging between 1.2-31.5. Moreover, the factorials were also unbalanced with respect to the number of crosses per parent line, which could range between 1 and 247 crosses per line. Some factorials such as Ra2 and Ra3 were very sparse, the percentage of realized hybrid combinations of all possible combinations being lower than 10%, while the factorial Wh1 was almost complete with almost 90% of realized hybrid combinations. The corn factorials Co1 and Co2 were created from the heterotic patterns of Flint and Dent (Technow et al., 2014; Schrag et al., 2018), while no heterotic pools were available for the wheat factorial Wh1 (Zhao et al., 2015) and the rapeseed factorials Ra1-3 (NPZ Innovation GmbH, personal communication). This is also reflected by the high contribution of SCA variance to total variance, and in the correlations of SCA and GCA with hybrid yield. As expected, we observed a higher relevance of SCA variance, and higher correlations of SCA with hybrid yield in the factorials for which no heterotic pools were available. Our initial research hypothesis was that ML algorithms might improve prediction accuracy in sparse, highly unbalanced factorials with a high relevance of SCA in comparison to GCA prediction based on a mixed linear model, and might even outperform GBLUP if genetic marker data is available.

### 4.1 Performance of ML algorithms based on parentage information and hybrid yields

We investigated three different scenarios, prediction without genotypic information, genomic prediction based on genotypic markers and a combination of both. In the first scenario, the only available information for predicting hybrid yields were the names of the hybrid parents, which we refer to as parentage information, and the hybrid yields from crosses with other parent lines from the factorial. For ANN, the parentage information was converted to binary variables indicating if a parent was used in a cross via one-hot encoding, all other algorithms used actual categorical variables with high cardinality. Using hybrid yields from crosses with other parent lines as predictors resulted in as many predictor variables as there were parent lines in the factorial, each variable being labeled with the name of a parent line and containing the information what yield was observed for the respective hybrid parents in a cross with this parent. Due to the unbalancedness and sparsity of the investigated factorials with respect to realized hybrid combinations, this led to predictor variables with a high percentage of missing values for rarely used parent lines, and numerous constant or almost constant variables for parent lines which were frequently used in crosses. Overall, the percentage of missing values in the predictor variables was way over 75% for most factorials in the cross validation splits, where all information from the validation set had to be removed from the training sets. As a consequence, some algorithms such as ANN did not converge for some of the factorials with this set of predictors, even if constant variables were dropped (**Figure S3**). For algorithms that converged, the use of hybrid yields as predictors considerably increased computation time, but never outperformed algorithms using parentage information only (**Figure S3**). We therefore focused our analysis on comparing ML algorithms based on parentage information to GCA prediction and GBLUP.

ML studies predicting yield without genotypic information have so far mostly focused on yield prediction for specific environments, using yield, parental and environmental information as well as management data as predictors. Shahhosseini et al. (2021) used GB, XGB and different ensembles for predicting corn yield of larger areas in the US corn belt with promising results. Khaki and Wang (2019) successfully designed a complex neural network for yield prediction in specific environments based on existing yield, environment and management data. The only other study using only parental information together with location and genetic clusters of the parents as input information to predict hybrid yield is the study of Khaki et al. (2020). However, this study was conducted using an exceptionally large data set of 294,128 hybrids provided by a large breeding company and a highly complex neural network approach. Babaie Sarijaloo et al. (2021) used the same dataset to compare different decision tree-based algorithms and neural networks and found XGB to be the best, due to its regularization and handling of sparse data. This effect may be weaker in our study since data of Babaie Sarijaloo et al. (2021) was even sparser than ours, with only 4% of all possible combinations available. No other study has to our knowledge investigated the use of ML algorithms with parentage information in small unbalanced factorials.

### 4.2 Performance of ML algorithms based on parentage in comparison to GCA prediction and GBLUP

GB was the best single ML algorithm for all data sets except Ra1 (best algorithm RF) and Co1 (best algorithm SVM, **Figure 1**). While ANN excel at complex tasks such as processing images, text or speech, tree-based methods such as GB, XGB and RF often perform better in tasks with structured, tabular data (Shwartz-Ziv and Armon, 2022) and thus might be especially suitable for predicting unbalanced factorials with potentially extreme under-/overrepresentation of some parent lines. Additionally, due to the slow and stepwise fitting process, GB has a low tendency to overfit the data (James et al., 2013). In factorials with a high relevance of SCA for yield, the superior performance of GB might be due to the fact that every new tree in a GB machine will fit to the residual of the previous trees, thus potentially improving the accuracy of the SCA component of prediction in comparison to linear model approaches. However, in the present study none of the single ML algorithms MF, SVM, RF, GB, ANN could outperform classical GCA prediction based on a mixed linear model to a meaningful extent (**Figure 1**), irrespective of the investigated data set.

The GB-SE was the only ML algorithm which performed better than GCA prediction in the factorials with the overall lowest prediction accuracies Ra1 and Ra2, increasing median prediction accuracy from 0.39 to 0.45, and from 0.48 to 0.54, respectively (**Figure 1**). For all other data sets, the performance of the GB-SE was equal to or only marginally better than GCA prediction. GBLUP was only investigated for the four data sets with available genotypic information (**Figure 1**). GBLUP resulted in median prediction accuracies equivalent to GCA prediction and the GB-SE in data sets Co1 and Co2. In data sets Ra1 and Wh1, GBLUP increased median prediction accuracy from 0.39 to 0.44 and from 0.71 to 0.74 in comparison to GCA prediction, and resulted in approximately equivalent prediction accuracies as the GB-SE. When considering the best 20 hybrids only, GB-SE resulted in the highest percental overlap between the predicted and observed 20 hybrids for factorials Ra1, Ra2, Ra3 and Co1, and GBLUP resulted in the highest overlap for factorials Wh1 and Co2 (**Figure S9**). Thus, GB-SE and GBLUP had the highest ability to predict the top 20 hybrids correctly, but the differences between both methods were small in all investigated data sets. Neither ML algorithms nor GBLUP did reduce the variation of prediction accuracies across cross validation splits in comparison to GCA prediction. Variation was generally low across cross validation splits for all investigated prediction models in factorial Wh1, which was an almost complete factorial, and in factorials Co1 and Co2 with a more balanced representation of parent groups and single parent lines (**Table 1**). Both factors most likely resulted in less extreme random splits for cross validation, in which many parent lines from the validation set were under-represented in the training set. From these findings, we conclude that the applicability of single ML algorithms in small unbalanced factorials is limited. GB-SEs might be a viable alternative where GCA prediction and GBLUP result in low prediction accuracies, or where no genotypic data is available, but the performance apparently depends on the structure and composition of the investigated factorials.

### 4.3 Effects of structure and composition of experimental data sets

While it is difficult to draw generally valid conclusions from a limited number of very diverse experimental data sets, we observed some trends: For the two corn factorials Co1 and Co2, all algorithms yielded high prediction accuracies ranging between 0.80 and 0.91 (**Figure 1**). Neither the ML algorithms nor GBLUP outperformed GCA prediction. In contrast to rapeseed and wheat, hybrid breeding with selection for GCA in corn has been established for decades, resulting in the two genetically diverse Flint and Dent pools. If heterotic pools exist, hybrid performance of heterotic traits is to a large extent explained by GCA effects, which can be accurately estimated from crosses with few testers from the opposite pool. This is also reflected in the high correlations of GCA with hybrid yield of 0.91 and 0.94 that we observed for these data sets (**Table 1**). The wheat data set Wh1 is a published data set from a study with the objective of establishing a new heterotic pattern (Zhao et al., 2015). Even though the two parent groups consequently form no heterotic pools, prediction accuracy with GCA effects was high with 0.71 (**Figure 1**). GBLUP and the GB-SE yielded slightly higher and comparable prediction accuracies with 0.74 and 0.72, respectively (**Figure 1**). In comparison to the other data sets, Wh1 is the smallest and most complete factorial with a percentage of realized hybrid combinations of 89.1% of all possible combinations (**Table 1**), which were evaluated in 11 environments (Zhao et al., 2015). The correlation of GCA with grain yield was comparatively high with 0.76, and higher than the correlation of SCA with grain yield (**Table 1**). We conclude that in crops with established heterotic pools such as corn, or in almost complete factorials with highly accurate phenotypic data, the potential of ML algorithms and even GBLUP for increasing prediction accuracy is limited, and hybrid yield can be efficiently predicted with GCA effects.

In the rapeseed data sets Ra1-Ra3, the overall levels of prediction accuracy as well as the potential for improvement with more complex algorithms varied considerably (**Figure 1**). In data sets Ra1 and Ra2, even though overall prediction accuracy was low, the GB-SE increased prediction accuracy by 14.5% from 0.39 to 0.45, and by 12.9% from 0.48 to 0.54 in comparison to GCA. GBLUP could, due to the unavailability of genotypic data, only be investigated in Ra1, and resulted in a prediction accuracy of 0.44 that was almost equivalent to the value of the GB-SE. In contrast to the results of Ra1 and Ra2, prediction accuracies in factorial Ra3 were comparatively high with values of 0.74 for GCA and 0.75 for the GB-SE. The three rapeseed factorials were to our knowledge not based on a heterotic pattern of the parent groups, but were pre-selected by the breeding company with the purpose to maximize hybrid yield (NPZ Innovation GmbH, personal communication). It is possible that parent groups for Ra3 were genetically more homogeneous within and more diverse between parent groups as was the case for Ra1 and Ra2. Factorials Ra1 and Ra2 are more sparse than Wh1 and Co2, with a percentage of realized hybrid combinations of 14.0% and 480 8.9% (**Table 1**), which might in part explain the overall low prediction accuracies. However, data set Ra3 is the sparsest of the investigated factorials, with a percentage of realized hybrid combinations of only 7.2%, and still resulted in prediction accuracies almost as high as the most complete factorial Wh1 (**Figure 1**). Moreover, Co1 with 11.9% of realized hybrid combinations is almost as sparse as Ra1 and Ra2 and still produced accurate GCA-based predictions and high prediction accuracies with the investigated ML algorithms.

### 4.4 Ratio of SCA to GCA effects

The major factors determining the overall level of prediction accuracy in the investigated factorials was τ describing the contribution of SCA variance to total variance, and the strength of the correlation of the SCA with hybrid yield. In the factorials Ra1 and Ra2 with overall low prediction accuracy, we observed very high correlations of hybrid yield with SCA of 0.93 and 0.88, while correlations of hybrid yield with GCA effects were lower in these data sets (**Table 1**). Conversely, in factorials with overall intermediate to high prediction accuracies in **Figure 1**, relevance of SCA variance and correlations of hybrid yield with SCA were always lower than correlations with GCA. This was most pronounced in the corn factorials Co1 and Co2 relying on the heterotic pattern of Flint and Dent. It also explains the high prediction accuracy observed in factorial Ra3 in comparison to factorials Ra1 and Ra2. In factorial Ra3, the correlation of hybrid yield with SCA amounted only to 0.69, while the correlation with GCA amounted to 0.92. The percentage of SCA variance in the total variance was low with 28% in factorial Ra3, and similar to the shares of 22% and 14% observed in factorials Co1 and Co2. In factorials Ra1 and Ra2, these proportions amounted to the highest with 67% and 51%of the total variance, respectively. The factorial Ra3 is exceptional, as in the absence of genetically diverse heterotic pools, hybrid performance is typically explained to a major extent by SCA. As the GB-SE was the best algorithm in factorials Ra1 andRa2 with high ratios of SCA variance to GCA variance, leading to considerable increases in prediction accuracy compared to GCA and equivalent values as GBLUP (**Figure 1**), we conclude that a potential field of application of ML algorithms in hybrid breeding programs is hybrid prediction in sparse unbalanced factorials with a high relevance of SCA effects.

### 4.5 Performance of ML algorithms with genotypic information

A major limitation of classical GCA prediction and the GB-SE based on parentage information compared to marker-based approaches is that hybrids for which only one or none of the parents have been tested before cannot be predicted. On the other hand, prediction accuracies are generally low for these so called type-1 and type-0 hybrids even with markers or more complex *omics* data (Zenke-Philippi et al., 2016, 511 Zenke-Philippi et al., 2017; Zhao et al., 2021). The potential to predict type-0 hybrids with marker-based ML in sufficiently large data sets remains to be investigated.

GBLUP did not increase prediction accuracy in comparison to classical GCA prediction based on phenotypic data and a mixed linear model in factorials Co1 and Co2 (**Figure 1**). In contrast, in factorials Ra1 and Wh1 GBLUP increased prediction accuracy from 0.39 to 0.44, and from 0.71 to 0.74 in comparison to GCA prediction. In all four factorials with available marker data, GBLUP resulted in comparable prediction accuracies as the GB-SE (**Figure 1**). From this, we conclude that a state-of-the-art GBLUP model is a perfectly suitable and efficient tool for predicting yield performance in sparse factorials with a high relevance of SCA. The GB-SE is a viable alternative to GBLUP, and can save the costs for genotyping the parent lines.

We expected that using marker data or a combination of marker data and parentage information with ML algorithms might further increase prediction accuracy in factorial Ra1, as genetic markers should contain much more detailed information on similarities between parent lines than parentage information alone, and might pick up non-additive effects and relationships between parent lines that are beyond the scope of the GBLUP model. ML algorithms should in theory be able to exploit also non-linear relationships and interactions between markers. We investigated four additional algorithms specifically tuned for marker data: XGB, RKHS, SVM and an XGB-SE (**Figure 2**). XGB and the XGB-SE resulted in the highest prediction accuracies of0.44 and 0.45, underlining again the superiority of GB machines for hybrid prediction in sparse unbalanced factorials.

Xu et al. (2021) have shown that combining phenotypic data of the parent lines with *omics* data improves the prediction accuracy. Similarly, other studies have combined genomic and pedigree information to outperform GBLUP (Sood et al., 2020). We therefore also considered a combined set of input variables including both marker data and parentage information for Ra1 and the best ML algorithms, XGB and XGB-SE. The prediction accuracies of the algorithms XGB-C and XGB-SE improved only slightly to 0.46 for both the single model and the ensemble (**Figure 2**). We therefore assume that with the investigated factorials, the ML algorithms mainly exploit the same information, i.e. the genetic similarity of the parents, and little additional information is gained when combining parentage information with marker data.

Other recent studies have observed that ML algorithms sometimes outperform the classical methods of genomic prediction based on mixed-model equations, but with an overall high variance in prediction accuracy across different traits and data sets (Azodi et al., 2019; Montesinos-López et al., 2019). Among the many ML algorithms available, those based on decision trees show promising results in many scenarios. Banerjee et al. (2020) found that tree-based algorithms such as RF, GB and XGB outperform classical genomic prediction algorithms. Westhues et al. (2021) used GB and XGB to improve yield prediction accuracy for genotypes across locations and years. Abdollahi-Arpanahi et al. (2020) also found that GB performs well when non-additive effects are important. Liang et al. (2021) achieved higher accuracies for three different animal data sets combining three different algorithms into an SE. Azodi et al. (2019) benchmarked several different algorithms on six different data sets with several traits each and also found that algorithm performance varied between data sets but an ensemble of different algorithms performed consistently equal or better than classical linear approaches. Yan et al. (2021) advocate the use of LightGBM, which is a more efficient type of GB due to building trees leave-wise instead of depth-wise. In their study, LightGBM also outperformed ridge regression BLUP for soybean and rice data. These results are in accordance with our finding that GB-SEs are more successful than single ML algorithms. However, in our study the use of XGB and an XGB-SE with genetic markers considerably increased computation time, but did not improve prediction accuracy compared to the GB-SE with parentage information (compare **Figure 2** with **Figure 1**). It is possible that the factorials were too small for effective pattern recognition in marker data, as the number of predictors was far higher than the number of hybrids. In factorial Ra1, 10880 markers were available after pre-processing for only 756 hybrids. In the case of comparatively small sparse factorials the positive effects of more detailed genetic information could be counter-balanced by increased dimensionality and noise, resulting in overfitting and spurious associations.

### 4.6 Hyperparameter optimization with random grid search

Selecting the best possible set of hyperparameters is crucial for maximizing prediction accuracy for a given data set. We used random grid search to test different hyperparameter combinations over a large hyperparameter space. In contrast to cartesian grid search, which tests all possible combinations of different values for each hyperparameter, the random grid search samples uniformly from the set of all possible hyperparameter combinations and stops when a user-specified criterion, e.g. a fixed number of models, is met. In most cases, random grid search performs as well as cartesian grid search while considerably reducing the computation time (Bergstra and Bengio, 2012). The frequency of how often a model was chosen as best or worst in a grid search for GB with parentage information is shown in **Figure 3**. For most of the investigated factorials, the best and worst grid search models accumulated on one or very few models. When considering the information given in **Table S2**, we can see that in the case where a few models have equal share in being the best, these models are similar in their hyperparameters. For example, the best combinations for factorial Ra2 all share the same value for the number of bins and an overall small sample rate. When only one or very few similar hyperparameter combinations repeatedly performed best in all cross validation splits of a given factorial, we assumed that these were very close to a theoretical ‘optimum’ hyperparameter combination for this scenario. If the selected hyperparameter combinations of the best models were far away from the optimum, we should observe more variation in the selected sets of hyperparameters across cross validation splits. We therefore conclude that the random grid search approach is a user-friendly, efficient and consistent approach for the identification of a suitable set of hyperparameters for all factorials investigated in the present study. We expect it to perform well in a wide range of crops and scenarios.

The fifty tuned GBs from the random grid search also formed the basis for building the GB-SE. We observed that some GB-SEs used all 50 models of the grid search (**Figure S10**). Thus, models that perform badly on their own might add some value when used within an ensemble, and a reduction in the number of investigated models for the random grid search might impair prediction accuracy. More efficient grid search methods such as hyperband search (Li et al., 2018b) and Bayesian optimization (Snoek et al., 2012) exist but have not yet been implemented in the software we used. Implementations of these might reduce the required computation time or find even better hyperparameter combinations. Galli et al. (2022) used an automated model training process to choose the best of 50 neural networks. This approach is even easier to apply than random grid search, with the downside that the hyperparameter space is always predefined and cannot be modified. Liang et al. (2022) propose a ‘tree-structured Parzen estimator’ that automatically tunes the hyperparameters. However, random grid search also performed well in this study.

### 4.7 Importance of different hyperparameters for ML algorithms

We focused our investigation on the hyperparameters for GB, which was the most successful single ML algorithm, and formed the basis for building the GB-SE. The most important hyperparameters with the greatest effect on reducing the MSE in GB with parentage information were the number of bins for partitioning the data before determining the tree’s best split point, and the depth of trees. Binning of factor levels for GB is also known as ‘histogram-based GB’. **Table 2** shows that for Ra1and Ra2 the number of bins is strongly correlated with the error. Setting a value for the number of bins that is smaller than the number of factor levels will group factor levels together into the specified number of groups (bins). For unordered nominal predictor variables as the parentage information investigated in the present study, these groups are somewhat arbitrary. Nevertheless, it seems that the number of bins has a large impact on the generalization error rate (Malohlava and Candel, 2022). As a tendency, reducing the number of bins for factors with high cardinality adds randomness to the splits in the decision trees, which seems to increase the generalizability of the model, while selecting a higher number of bins increases model fit to the training data and can lead to overfitting. When taking the ridge line plots in **Figure S4** into account, it seems that the relationship between the number of bins and the error is not linear. Too many as well as too few bins both increase the error. Selecting a medium number of bins had a positive effect on the prediction accuracy, especially for data sets Ra1 and Ra2 with overall low prediction accuracy. This is also reflected in **Table S2**, where we listed the hyperparameters for the best models investigated in the random grid search. As trees for GB always need to be trained sequentially, model training is slow in comparison to other models such as RF, for which trees can be trained in parallel. As a desirable side effect, binning will considerably speed up model training. We therefore recommend tuning of the number of bins if many parents, i.e. many factor levels, have to be included in the model in order to increase efficiency and performance of GB. It remains to be investigated if ordered binning, e.g. by clustering genetically similar parents together, can further increase prediction accuracy.

With respect to the depth of the decision trees, we observed that the factorials Ra1-3 required deeper trees than the factorials Wh1, Co1 and Co2 (**Table S2**). Usually the depth of the trees is related to the complexity of the task. We observed high accuracies of GCA effects for factorials Wh1, Co1 and Co2, indicating that hybrid performance can be accurately represented by the additive parent GCA effects. Consequently, fewer splits per tree are also needed in GB. For the rapeseed factorials Ra1 and Ra2 with a high relevance of SCA for hybrid yield, deeper trees might be able to capture non-additive SCA effects. We saw little room for reducing the computation time of the random grid search by a more limited search space when initially looking for the best model for the investigated factorials, since hyperparameters from the upper and the lower end of our search space were considered best in some cases.

### 4.8 Implementation of machine learning algorithms in hybrid breeding programs

The required know-how and computational power is a major obstacle for the implementation of more complex ML algorithms in hybrid breeding programs. In particular, feature selection, model implementation in languages such as Python or Julia, the tuning of hyperparameters and the composition of ensembles of models are tasks which may seem daunting for breeders without solid background in data science. Our study has shown that successful prediction of hybrid yield does not necessarily rely on very large datasets and expert-designed models. Here, we present a fairly automated random grid search approach for building GB-SEs with the parentage information as a predictor, implemented in a user-friendly software package with an R interface. The well-documented Rcode for our procedure is available in the **supplementary material**, and can be tested with the publicly available experimental data sets investigated in our study. Many breeders are already familiar with analysis of field trials and genomic selection in R, and can thus easily adapt the code for their own breeding programs and purposes.

We expect that ML algorithms will only be widely implemented in practical breeding programs if they offer advantages over well-established prediction models such as GCA prediction and GBLUP in real-life data sets from ongoing breeding programs. In the present study, we observed potential for ML algorithms in comparatively small, sparse unbalanced factorials with high relevance of SCA effects. The GB-based SE with parentage information increased prediction accuracy considerably in these specific factorials in comparison to classical GCA prediction, and resulted in equivalent prediction accuracies in comparison to GBLUP for all other investigated factorials. Moreover, the random grid search approach for tuning the basic GBs delivered consistent sets of hyperparameters across cross-validation splits in reasonable computation time. In comparison to GBLUP and other marker-based approaches, the simple use of parents as predictors can also save the cost of genotyping. We therefore expect that our suggested procedure is applicable in wide range of crops and breeding programs, and can be considered as an alternative to GBLUP.

For the present study, we decided to test the ML algorithms in crops of major commercial importance, because high-quality genotypic and phenotypic data was easily accessible, and we were able to compare long-established hybrid breeding programs with high relevance of GCA with newly established programs with high relevance of SCA. However, we do not expect that large hybrid breeding programs with well-established genomic selection pipelines represent the main field of application for our method. A recent review on hybrid breeding from the perspective of commercial breeders has pointed out that breeding targets shift and change over time, and that older cultivars and selection candidates form an important secondary breeding pool that might contain useful variation for new traits of interest (Steeg et al., 2022). One potential application of the GB-SE based on parentage information might be to pre-screen older field trials without *ad-hoc* available genotypic data and pre-select interesting candidates for such novel breeding targets for future testing. The same paper has also pointed out that on a world-wide level about 6000 plants are currently under cultivation. Only about 50 of those are currently bred as hybrids, exploiting hybrid vigor and other advantages for both commercial breeders and consumers. This suggests great future potential for establishment of hybrid breeding also in crops of minor commercial importance. New hybrid breeding programs have been established in crops such as guava, onion, eggplant, potato, triticale etc. Such newly set-up hybrid programs are often characterized by a high relevance of SCA. Many of these minor crops with high relevance for food diversity are bred in small breeding programs with very low budget, restricted number of field plots, and very limited staff. Not for all of these breeding programs high-throughput genotyping is readily available at the moment. For these breeding programs, the GB-SE with parentage information could provide a viable short-term alternative to genomic prediction.

## Data availability statement

Publicly available datasets were analyzed in this study. The phenotypic and genotypic data of experiments Co1 and Co2 and the phenotypic data for experiment Wh1 have been published as **Supplementary Materials** to the research articles cited in the material and methods section. The genotypic data for experiment Wh1 is available at https://datadryad.org/stash/dataset/doi:10.5061/dryad.461nc. Data sets Ra1-3 were provided by NPZ Innovation GmbH and are confidential.

## Author contributions

EH and PH conceived the study. AA and TK provided the phenotypic and genotypic data of experiments Ra1-3. PH compiled the phenotypic and genotypic data of experiments Wh1, Co1 and Co2 from public data sources. PH pre-processed and analyzed the phenotypic and genotypic data, fitted the prediction models and conducted the predictions. PH and EH wrote the manuscript with further input from MF. All authors contributed to the article and approved the submitted version.

## References

[B1] Abbas Q. Ibrahim M. E. Jaffar M. A. (2019). A comprehensive review of recent advances on deep vision systems. Artif. Intell. Rev. 52, 39–76. doi: 10.1007/s10462-018-9633-3

[B2] Abdollahi-Arpanahi R. Gianola D. Peñagaricano F. (2020). Deep learning versus parametric and ensemble methods for genomic prediction of complex phenotypes. Genet. Selection Evol. 52, 12. doi: 10.1186/s12711-020-00531-z PMC7038529 32093611

[B3] Albrecht T. Wimmer V. Auinger H.-J. Erbe M. Knaak C. Ouzunova M.. (2011). Genome-based prediction of testcross values in maize. Theor. Appl. Genet. 123, 339–350. doi: 10.1007/s00122-011-1587-7 21505832

[B4] Azodi C. B. Bolger E. McCarren A. Roantree M. de los Campos G. Shiu S.-H. (2019). Benchmarking parametric and machine learning models for genomic prediction of complex traits. G3: Genes Genomes Genet. 9, 3691–3702. doi: 10.1534/g3.119.400498 PMC6829122 31533955

[B5] Babaie Sarijaloo F. Porta M. Taslimi B. Pardalos P. M. (2021). Yield performance estimation of corn hybrids using machine learning algorithms. Artif. Intell. Agric. 5, 82–89. doi: 10.1016/j.aiia.2021.05.001

[B6] Banerjee R. Marathi B. Singh M. (2020). Efficient genomic selection using ensemble learning and ensemble feature reduction. J. Crop Sci. Biotechnol. 23, 311–323. doi: 10.1007/s12892-020-00039-4

[B7] Bates D. Mächler M. Bolker B. Walker S. (2015). Fitting linear mixed-effects models using lme4. J. Stat. Software 67, 1-48. doi: 10.18637/jss.v067.i01

[B8] Bergstra J. Bengio Y. (2012). Random search for hyper-parameter optimization. J. Mach. Learn. Res. 13, 281–305.

[B9] Bischl B. Lang M. Kotthoff L. Schiffner J. Richter J. Studerus E.. (2016). Mlr: machine learning in r. J. Mach. Learn. Res. 17, 1–5.

[B10] Bischl B. Lang M. Schratz P. (2020) Parallelmap: unified interface to parallelization back-ends r package version 1.5.0. Available at: https://cran.r-project.org/package=parallelMap.

[B11] Bishop C. M. (2006). Pattern recoginiton and machine learning (New York, USA: Springer-Verlag New York).

[B12] Breiman L. (1996). Stacked regressions. Mach. Learn. 24, 49–64. doi: 10.1023/A:1018046112532

[B13] Breiman L. (2001). Random forests. Mach. Learn. 45, 5–32. doi: 10.1023/A:1010933404324

[B14] Butler K. T. Davies D. W. Cartwright H. Isayev O. Walsh A. (2018). Machine learning for molecular and materials science. Nature 559, 547–555. doi: 10.1038/s41586-018-0337-2 30046072

[B15] Chen T. Guestrin C. (2016). “Xgboost: a scalable tree boosting system,” in Proceedings of the 22nd ACM SIGKDD international conference on knowledge discovery and data mining (New York, NY, USA: Association for Computing Machinery). doi: 10.1145/2939672.2939785

[B16] Chen T. He T. Benesty M. Khotilovich V. Tang Y. Cho H.. (2022) Xgboost: extreme gradient boosting r package version 1.6.0.1. Available at: https://CRAN.R-project.org/package=xgboost.

[B17] Cortes C. Vapnik V. (1995). Support-vector networks. Mach. Learn. 20, 273–297. doi: 10.1007/bf00994018

[B18] Covarrubias-Pazaran G. (2016). Genome assisted prediction of quantitative traits using the r package sommer. PloS One 11, 1–15. doi: 10.1371/journal.pone.0156744 PMC4894563 27271781

[B19] Covarrubias-Pazaran G. (2018). Software update: moving the r package sommer to multivariate mixed models for genome-assisted prediction. biorxv. 354639. doi: 10.1101/354639

[B20] Cuevas J. Montesinos-López O. Juliana P. Guzmán C. Pérez-Rodríguez P. González-Bucio J.. (2019). Deep kernel for genomic and near infrared predictions in multi-environment breeding trials. G3: Genes Genomes Genet. 9, 2913–2924. doi: 10.1534/g3.119.400493 PMC6723142 31289023

[B21] Dargan S. Kumar M. Ayyagari M. R. Kumar G. (2020). A survey of deep learning and its applications: a new paradigm to machine learning. Arch. Comput. Methods Eng. 27, 1071–1092. doi: 10.1007/s11831-019-09344-w

[B22] Domingos P. (2012). A few useful things to know about machine learning. Commun. ACM 55, 78–87. doi: 10.1145/2347736.2347755

[B23] Endelman J. B. Jannink J.-L. (2012). Shrinkage estimation of the realized relationship matrix. G3: Genes Genomes Genet. 2, 1405–1413. doi: 10.1534/g3.112.004259 PMC3484671 23173092

[B24] Friedman J. H. (2001). Greedy function approximation: a gradient boosting machine. Ann. Stat 29, 1189–1232. doi: 10.1214/aos/1013203451

[B25] Gabur I. Simioniuc D. P. Snowdon R. J. Cristea D. (2022). Machine learning applied to the search for nonlinear features in breeding populations. Front. Artif. Intell. 5. doi: 10.3389/frai.2022.876578 PMC9164111 35669178

[B26] Galli G. Sabadin F. Yassue R. M. Galves C. Carvalho H. F. Crossa J.. (2022). Automated machine learning: a case study of genomic “image-based” prediction in maize hybrids. Front. Plant Sci. 13. doi: 10.3389/fpls.2022.845524 PMC8936805 35321444

[B27] Gillberg J. Marttinen P. Mamitsuka H. Kaski S. (2019). Modelling gxe with historical weather information improves genomic prediction in new environments. Bioinformatics 35, 4045–4052. doi: 10.1093/bioinformatics/btz197 30977782 PMC6792123

[B28] Goodfellow I. Bengio Y. Courville A. (2016). Deep learning (Cambridge, MA, USA: MIT Press). Available at: http://www.deeplearningbook.org.

[B29] Gowda M. Zhao Y. Würschum T. Longin C. F. Miedaner T. Ebmeyer E.. (2014). Relatedness severely impacts accuracy of marker-assisted selection for disease resistance in hybrid wheat. Heredity 112, 552–561. doi: 10.1038/hdy.2013.139 24346498 PMC3998782

[B30] Hallauer A. R. Carena M. J. Miranda Filho J. B. (2010). Quantitative genetics in maize breeding (New York, USA: Springer).

[B31] Hastie T. Tibshirani R. Friedman J. (2009). The elements of statistical learning (New York, USA: Springer). doi: 10.1007/978-0-387-84858-7

[B32] Hofheinz N. Borchardt D. Weissleder K. Frisch M. (2012). Genome-based prediction of test cross performance in two subsequent breeding cycles. Theor. Appl. Genet. 125, 1639–1645. doi: 10.1007/s00122-012-1940-5 22814724

[B33] James G. Witten D. Hastie T. Tibshirani R. (2013). An introduction to statistical learning (New York, USA: Springer). doi: 10.1007/978-1-4614-7138-7

[B34] Karatzoglou A. Hornik K. Smola A. Zeileis A. (2004). Kernlab - an s4 package for kernel methods in r. J. Stat. Software 11, 1–20. doi: 10.18637/jss.v011.i09

[B35] Khaki S. Khalilzadeh Z. Wang L. (2020). Predicting yield performance of parents in plant breeding: a neural collaborative filtering approach. PLoS One 15, e0233382. doi: 10.1371/journal.pone.0233382 32437473 PMC7241707

[B36] Khaki S. Wang L. (2019). Crop yield prediction using deep neural networks. Front. Plant Sci. 10. doi: 10.3389/fpls.2019.00621 PMC6540942 31191564

[B37] Koren Y. Bell R. Volinsky C. (2009). Matrix factorization techniques for recommender systems. Computer 42, 30–37. doi: 10.1109/MC.2009.263

[B38] LeDell E. Gill N. Aiello S. Fu A. Candel A. Click C.. (2020). h2o: r interface for the “h2o” scalable machine learning platform r package version 3.32.0.3.

[B39] Lenth R. (2021) Emmeans: estimated marginal means, aka least-squares means r package version 1.2.3. Available at: https://cran.r-project.org/package=emmeans.

[B40] Li L. Jamieson K. DeSalvo G. Rostamizadeh A. Talwalkar A. (2018b). Hyperband: a novel bandit-based approach to hyperparameter optimization. J. Mach. Learn. Res. 18, 1–52. doi: 10.3389/fgene.2018.00237

[B41] Li B. Zhang N. Wang Y.-G. George A. W. Reverter A. Li Y. (2018a). Genomic prediction of breeding values using a subset of snps identified by three machine learning methods. Front. Genet. 9. doi: 10.3389/fgene.2018.00237 PMC6039760 30023001

[B42] Liang M. An B. Li K. Du L. Deng T. Cao S.. (2022). Improving genomic prediction with machine learning incorporating tpe for hyperparameters optimization. Biology. 11, 1647. doi: 10.3390/biology11111647 PMC9688023 36421361

[B43] Liang M. Chang T. An B. Duan X. Du L. Wang X.. (2021). A stacking ensemble learning framework for genomic prediction. Front. Genet. 12. doi: 10.3389/fgene.2021.600040 PMC7969712 33747037

[B44] Ma W. Qiu Z. Song J. Li J. Cheng Q. Zhai J.. (2018). A deep convolutional neural network approach for predicting phenotypes from genotypes. Planta 248, 1307–1318. doi: 10.1007/s00425-018-2976-9 30101399

[B45] Malohlava M. Candel A. (2022). Gradient boosting machine with h2o (Mountain View, CA, USA: H2O.ai Inc). Available at: https://docs.h2o.ai/h2o/latest-stable/h2o-docs/booklets/GBMBooklet.pdf.

[B46] Melchinger A. E. Gumber R. K. (1998). “Chap. 3,” in Overview of heterosis and heterotic groups in agronomic crops (Madison, WI, USA: John Wiley & Sons, Ltd), 29–44.

[B47] Mohanty S. P. Hughes D. P. Salathé M. (2016). Using deep learning for image-based plant disease detection. Front. Plant Sci. 7. doi: 10.3389/fpls.2016.01419 PMC5032846 27713752

[B48] Montesinos-López O. A. Martín-Vallejo J. Crossa J. Gianola D. Hernández-Suárez C. M. Montesinos-López A.. (2019). A benchmarking between deep learning, support vector machine and bayesian threshold best linear unbiased prediction for predicting ordinal traits in plant breeding. G3: Genes Genomes Genet. 9, 601–618. doi: 10.1534/g3.118.200998 PMC6385991 30593512

[B49] Montesinos-López O. A. Montesinos-López A. Crossa J. Gianola D. Hernández-Suárez C. M. Martín-Vallejo J. (2018a). Multi-trait, multi-environment deep learning modeling for genomic-enabled prediction of plant traits. G3: Genes Genomes Genet. 8, 3829–3840. doi: 10.1534/g3.118.200728 PMC6288830 30291108

[B50] Montesinos-López O. A. Montesinos-López A. Crossa J. Montesinos-López J. C. Mota-Sanchez D. Estrada-González F.. (2018b). Prediction of multiple-trait and multiple-environment genomic data using recommender systems. G3: Genes Genomes Genet. 8, 131–147. doi: 10.1534/g3.117.300309 PMC5765342 29097376

[B51] Montesinos-López O. A. Montesinos-López A. Pérez-Rodríguez P. Barrón-López J. A. Martini J. W. Fajardo-Flores S. B.. (2021). A review of deep learning applications for genomic selection. BMC Genomics 22, 19. doi: 10.1186/s12864-020-07319-x 33407114 PMC7789712

[B52] Nagasubramanian K. Jones S. Sarkar S. Singh A. K. Singh A. Ganapathysubramanian B. (2018). Hyperspectral band selection using genetic algorithm and support vector machines for early identification of charcoal rot disease in soybean stems. Plant Methods 14, 86. doi: 10.1186/s13007-018-0349-9 30305840 PMC6169113

[B53] Nagasubramanian K. Jones S. Singh A. K. Sarkar S. Singh A. Ganapathysubramanian B. (2019). Plant disease identification using explainable 3d deep learning on hyperspectral images. Plant Methods 15, 98. doi: 10.1186/s13007-019-0479-8 31452674 PMC6702735

[B54] Perez P. de los Campos G. (2014). Genome-wide regression and prediction with the bglr statistical package. Genetics 198, 483–495. doi: 10.1534/genetics.114.164442 25009151 PMC4196607

[B55] Philipp N. Liu G. Zhao Y. He S. Spiller M. Stiewe G.. (2016). Genomic prediction of barley hybrid performance. Plant Genome 9, plantgenome 2016–02. doi: 10.3835/plantgenome2016.02.0016 27898835

[B56] Probst P. Boulesteix A.-L. Bischl B. (2019). Tunability: importance of hyperparameters of machine learning algorithms. J. Mach. Learn. Res. 20, 1–32.

[B57] Qiu Y. Cortes D. Lin C.-J. Juan Y.-C. Chin W.-S. Zhuang Y.. (2021). Recosystem: recommender system using matrix factorization.

[B58] R Core Team (2022). R: a language and environment for statistical computing (Vienna, Austria: R Foundation for Statistical Computing).

[B59] Schrag T. A. Westhues M. Schipprack W. Seifert F. Thiemann A. Scholten S.. (2018). Beyond genomic prediction: combining different types of omics data can improve prediction of hybrid performance in maize. Genetics 208, 1373–1385. doi: 10.1534/genetics.117.300374 29363551 PMC5887136

[B60] Schulthess A. W. Zhao Y. Reif J. C. (2017). Genomic selection in hybrid breeding (Cham: Springer International Publishing), 149–183.

[B61] Shahhosseini M. Hu G. Huber I. Archontoulis S. V. (2021). Coupling machine learning and crop modeling improves crop yield prediction in the us corn belt. Sci. Rep. 11, 1–15. doi: 10.1038/s41598-020-80820-1 33452349 PMC7810832

[B62] Shwartz-Ziv R. Armon A. (2022). Tabular data: deep learning is not all you need. Inf. Fusion 81, 84–90. doi: 10.1016/j.inffus.2021.11.011

[B63] Snoek J. Larochelle H. Adams R. P. (2012). “Practical bayesian optimization of machine learning algorithms,” in Advances in neural information processing systems, F. Pereira and C.J. Burges and L. Bottou and K.Q. Weinberger (Red Hook, NY, USA: Curran Associates Inc), 25, 2951–2959. Available at: https://proceedings.neurips.cc/paper_files/paper/2012/file/05311655a15b75fab86956663e1819cd-Paper.pdf.

[B64] Sood S. Lin Z. Caruana B. Slater A. T. Daetwyler H. D. (2020). Making the most of all data: combining non-genotyped and genotyped potato individuals with hblup. Plant Genome 13, e20056. doi: 10.1002/tpg2.20056 33217206 PMC12807232

[B65] Stahl A. Pfeifer M. Frisch M. Wittkop B. Snowdon R. J. (2017). Recent genetic gains in nitrogen use efficiency in oilseed rape. Front. Plant Sci. 8, 963. doi: 10.3389/fpls.2017.00963 28638399 PMC5461335

[B66] Steeg E. Struik P. Visser R. Lindhout P. (2022). Crucial factors for the feasibility of commercial hybrid breeding in food crops. Nat. Plants 8, 1–11. doi: 10.1038/s41477-022-01142-w 35513713

[B67] Stuber C. W. Cockerham C. C. (1966). Gene effects and variances in hybrid populations. Genetics 54, 1279–1286. doi: 10.1093/genetics/54.6.1279 17248353 PMC1211293

[B68] Technow F. Riedelsheimer C. Schrag T. A. Melchinger A. E. (2012). Genomic prediction of hybrid performance in maize with models incorporating dominance and population specific marker effects. Theor. Appl. Genet. 125, 1181–1194. doi: 10.1007/s00122-012-1905-8 22733443

[B69] Technow F. Schrag T. A. Schipprack W. Bauer E. Simianer H. Melchinger A. E. (2014). Genome properties and prospects of genomic prediction of hybrid performance in a breeding program of maize. Genetics 197, 1343–1355. doi: 10.1534/genetics.114.165860 24850820 PMC4125404

[B70] Van Der Laan M. J. Polley E. C. Hubbard A. E. (2007). “Super learner,” in Statistical applications in genetics and molecular biology. 6, 25. doi: 10.2202/1544-6115.1309 17910531

[B71] Washburn J. D. Cimen E. Ramstein G. Reeves T. O’Briant P. McLean G.. (2021). Predicting phenotypes from genetic, environment, management, and historical data using cnns. Theor. Appl. Genet. 134, 3997–4011. doi: 10.1007/s00122-021-03943-7 34448888

[B72] Westhues C. C. Mahone G. S. da Silva S. Thorwarth P. Schmidt M. Richter J. C.. (2021). Prediction of maize phenotypic traits with genomic and environmental predictors using gradient boosting frameworks. Front. Plant Sci. 12. doi: 10.3389/fpls.2021.699589 PMC8647909 34880880

[B73] Xu Y. Zhao Y. Wang X. Ma Y. Li P. Yang Z.. (2021). Incorporation of parental phenotypic data into multi-omic models improves prediction of yield-related traits in hybrid rice. Plant Biotechnol. J. 19, 261–272. doi: 10.1111/pbi.13458 32738177 PMC7868986

[B74] Yan J. Xu Y. Cheng Q. Jiang S. Wang Q. Xiao Y.. (2021). Lightgbm: accelerated genomically designed crop breeding through ensemble learning. Genome Biol. 22, 271. doi: 10.1186/s13059-021-02492-y 34544450 PMC8451137

[B75] Zenke-Philippi C. Frisch M. Thiemann A. Seifert F. Schrag T. Melchinger A. E.. (2017). Transcriptome-based prediction of hybrid performance with unbalanced data from a maize breeding programme. Plant Breed. 136, 331–337. doi: 10.1111/pbr.12482

[B76] Zenke-Philippi C. Thiemann A. Seifert F. Schrag T. Melchinger A. E. Scholten S.. (2016). Prediction of hybrid performance in maize with a ridge regression model employed to dna markers and mrna transcription profiles. BMC Genomics 17, 262. doi: 10.1186/s12864-016-2580-y 27025377 PMC4812617

[B77] Zhao Y. Li Z. Liu G. Jiang Y. Maurer H. P. Würschum T.. (2015). Genome-based establishment of a high-yielding heterotic pattern for hybrid wheat breeding. Proc. Natl. Acad. Sci. U.S.A. 112, 15624–15629. doi: 10.1073/pnas.1514547112 26663911 PMC4697414

[B78] Zhao Y. Thorwarth P. Jiang Y. Philipp N. Schulthess A. W. Gils M.. (2021). Unlocking big data doubled the accuracy in predicting the grain yield in hybrid wheat. Sci. Adv. 7, eabf9106. doi: 10.1126/sciadv.abf9106 34117061 PMC8195483

