# Supplementary material for: Stacked ensembles on basis of parentage information can predict hybrid performance with an accuracy comparable to marker-based GBLUP
Source: Front Plant Sci. 2023 Jul 21;14:1178902. doi: 10.3389/fpls.2023.1178902 (PMC10401275; doi:10.3389/fpls.2023.1178902)
Supplement: Supplementary file 1 [file DataSheet_1.pdf]

Supplementary Material: Stacked ensembles on basis of parentage information can predict hybrid performance with an accuracy comparable to marker-based GBLUP

1 SUPPLEMENTARY TABLES AND FIGURES

1.1 Figures

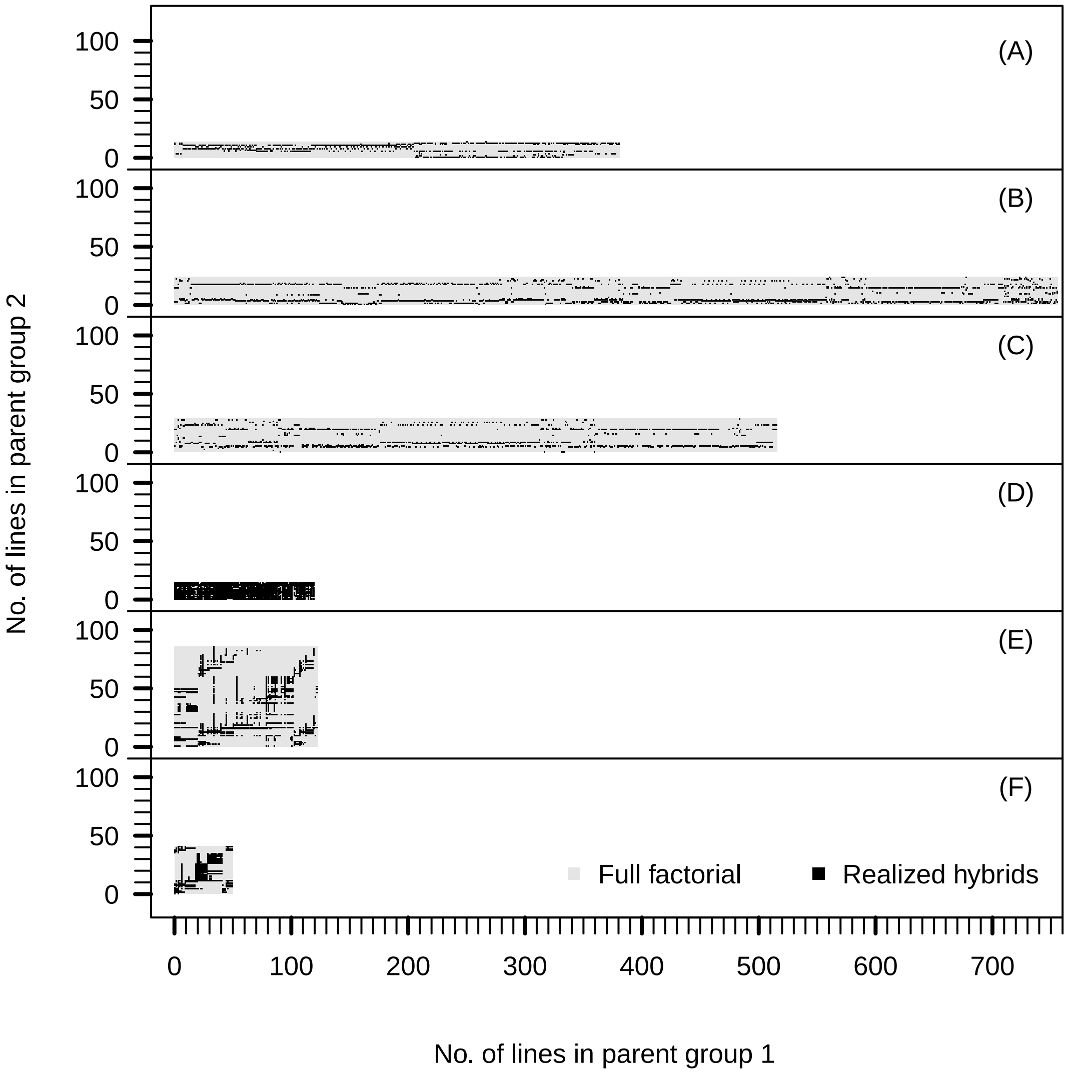

Figure S1. Crossing matrices for every experimental dataset indicating the size of the parent groups as well as the number of realized hybrid combinations. Black tiles represent realized hybrid crosses. The factorials are displayed on the same scales to highlight the differences between datasets with regard to size, unbalancedness in size of parent groups and sparsity. Datasets: **(A)**: Ra1, 14 × 381; **(B)**: Ra2, 24 × 756; **(C)**: Ra3, 29 × 516; **(D)**: Wh1, 15 × 120; **(E)**: Co1, 86 × 123; **(F)**: Co2, 50 × 41.

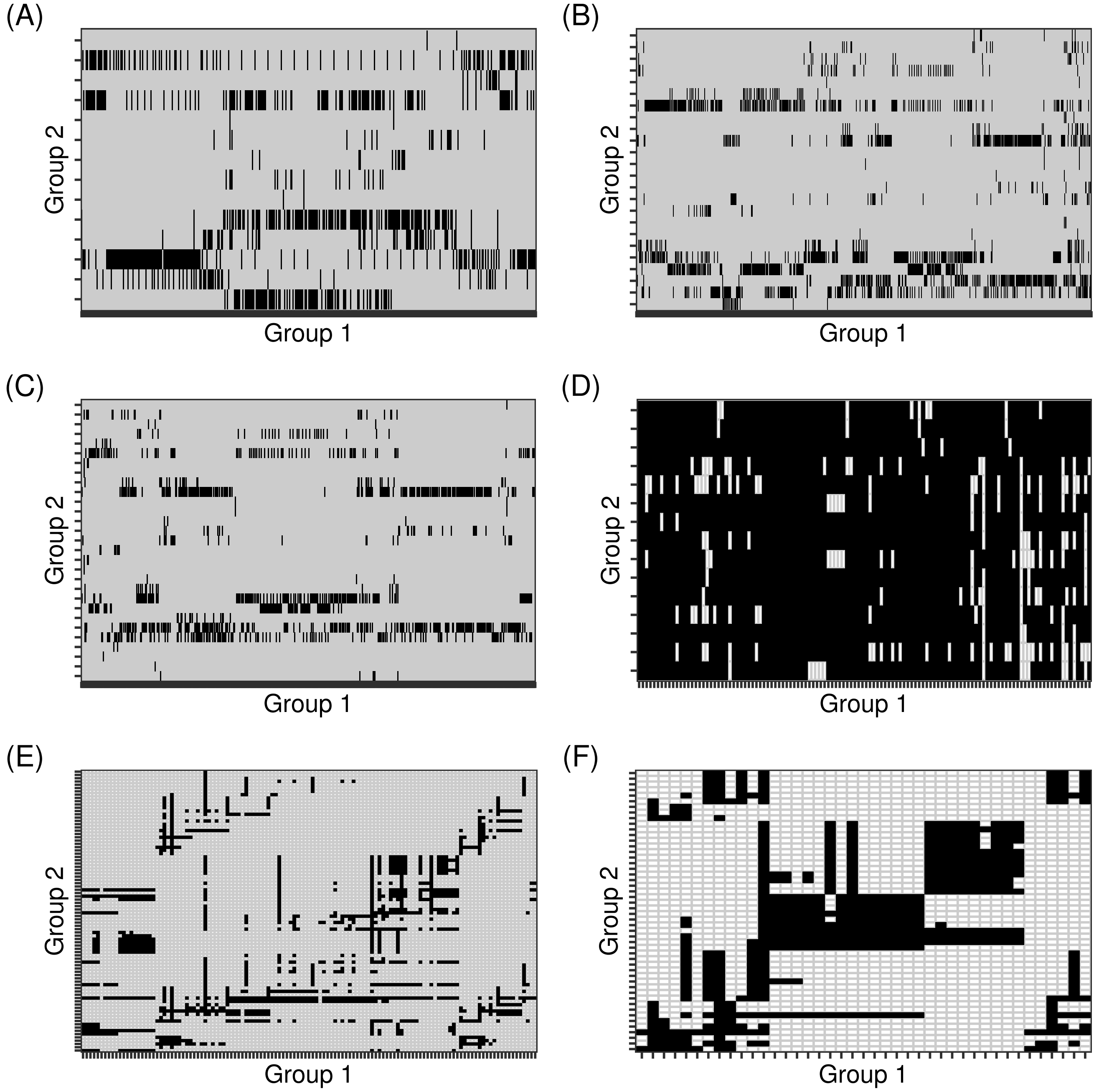

Figure S2. Crossing matrices of the individual datasets indicating the sparsity/completeness of the factorial. Black tiles represent realized crosses. Tick marks on both axes represent the lines from the respective group. Enlarged representation of Figure S1. Datasets: **(A)**: Ra1, 14 × 381; **(B)**: Ra2, 24 × 756; **(C)**: Ra3, 29 × 516; **(D)**: Wh1, 15 × 120; **(E)**: Co1, 86 × 123; **(F)**: Co2, 50 × 41.

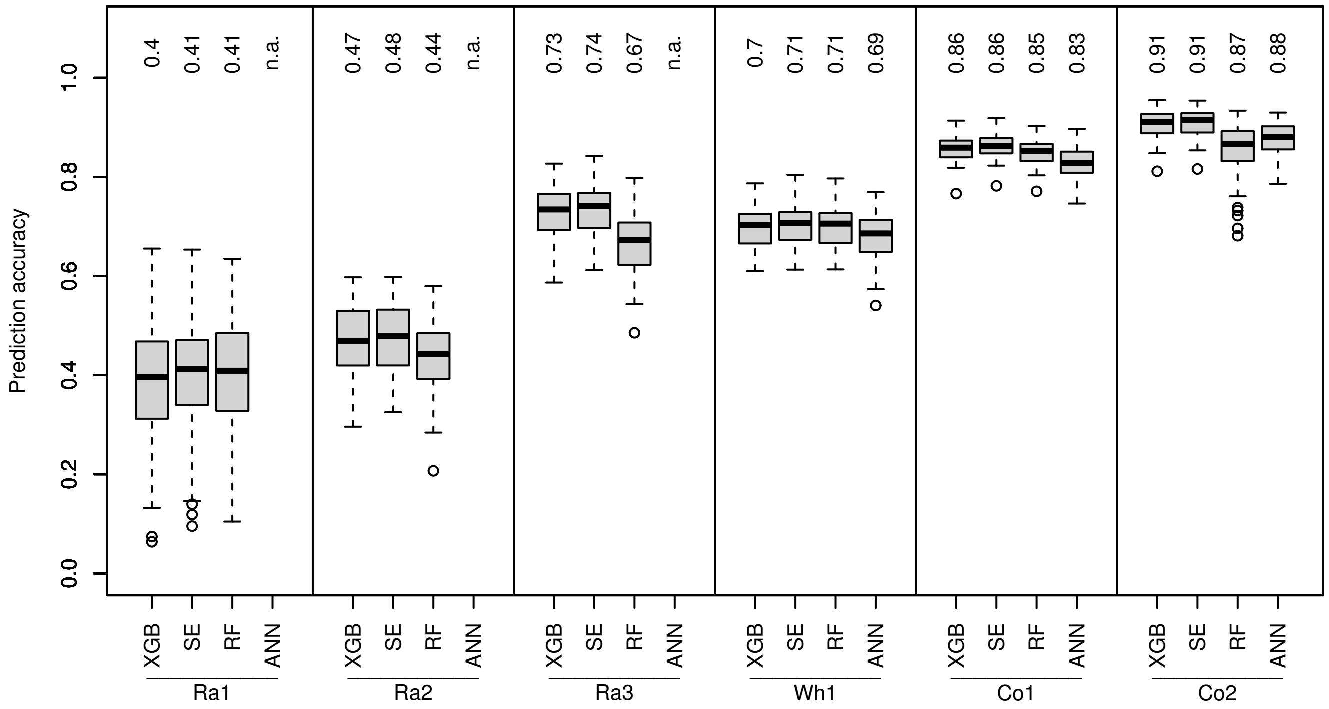

Figure S3. Boxplots of observed prediction accuracies for six different datasets (Ra1, Ra2, Ra3, Wh1, Co1, Co2) using four different methods (XGB, SE, RF, ANN) and yield of all other realized crosses of the parents of a specific hybrid as input features. Median prediction accuracy is displayed above each boxplot. As an alternative to the parentage information approach which uses parent names as input variables, we used the yield of all other realized crosses of the parents of a specific hybrid as the input features to predict its yield. This set of input features increased computation time, but never outperformed prediction with nominal parent factor levels. Results of ANN missing (n.a.) for datasets Ra1, Ra2 and Ra3 because models did not converge during training.

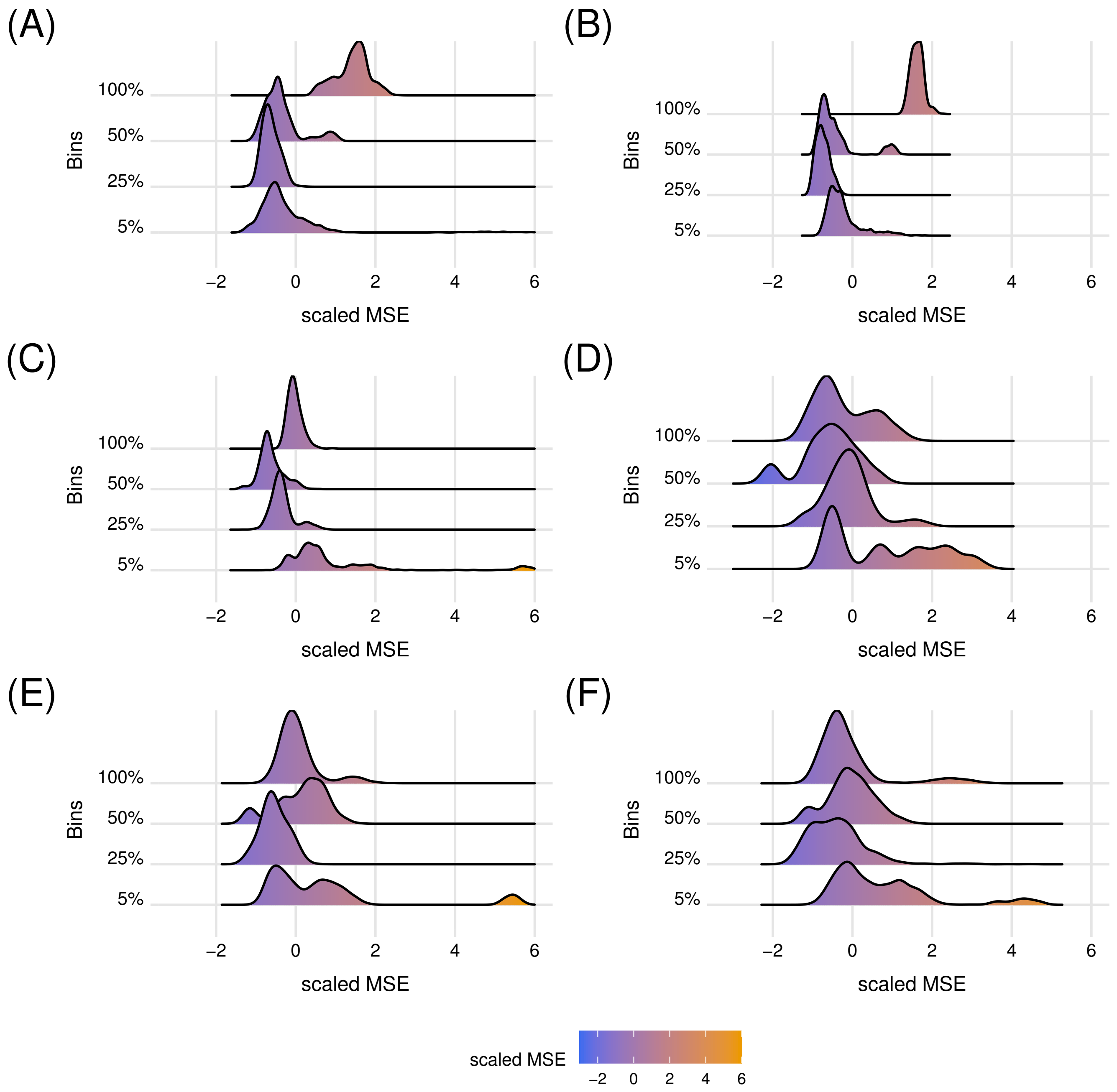

Figure S4. Distribution of the scaled mean squared error (MSE) per hyperparameter level of number of bins used for categories. Error of every grid search model was scaled by subtracting the mean and dividing it by the standard deviation of the respective cross validation split. Datasets: **(A)**: Ra1, **(B)**: Ra2, **(C)**: Ra3, **(D)**: Wh1, **(E)**: Co1, **(F)**: Co2

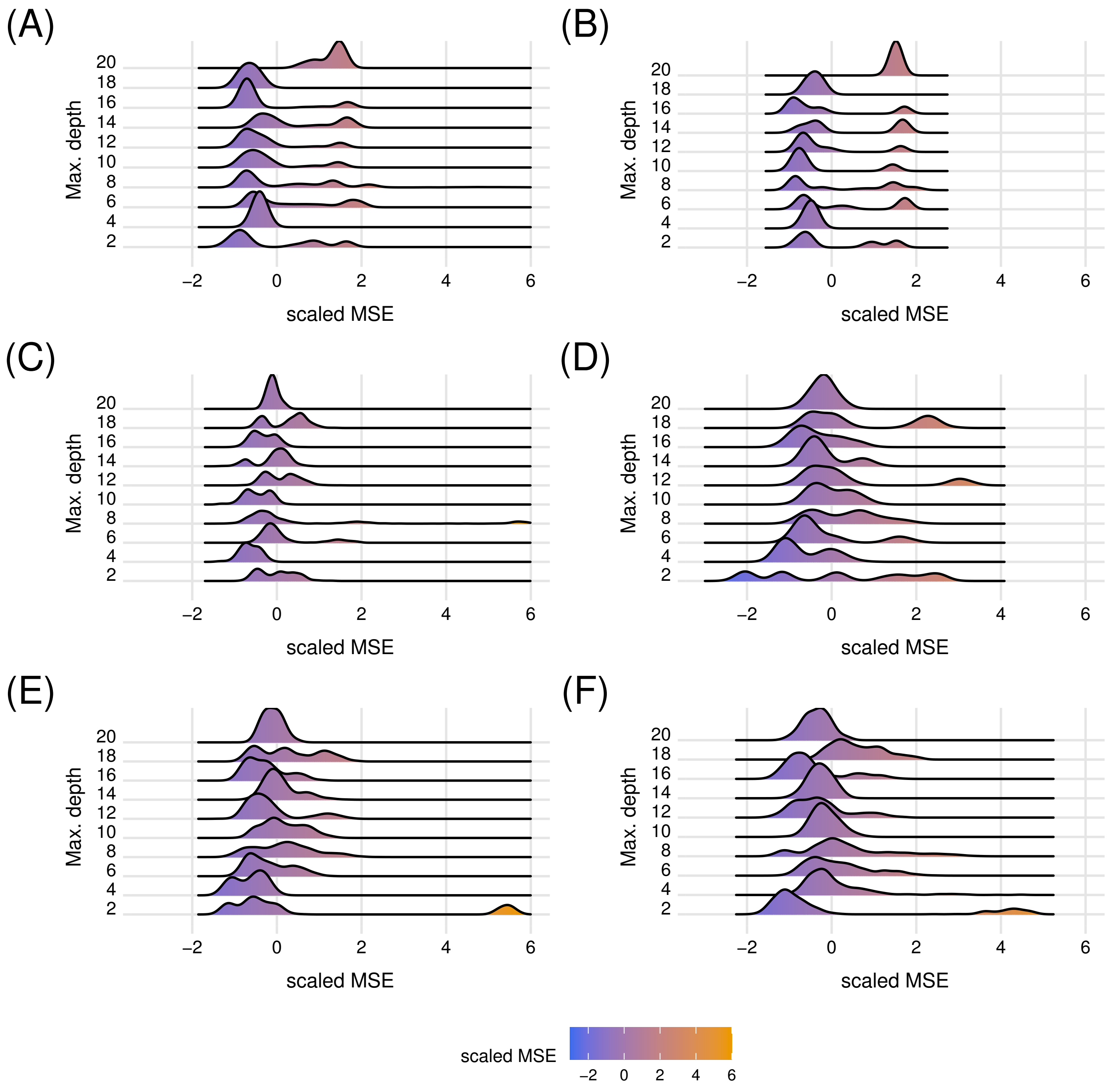

Figure S5. Distribution of the scaled mean squared error (MSE) per hyperparameter level of maximum depth of trees. Error of every grid search model was scaled by subtracting the mean and dividing it by the standard deviation of the respective cross validation split. Datasets: **(A)**: Ra1, **(B)**: Ra2, **(C)**: Ra3, **(D)**: Wh1, **(E)**: Co1, **(F)**: Co2

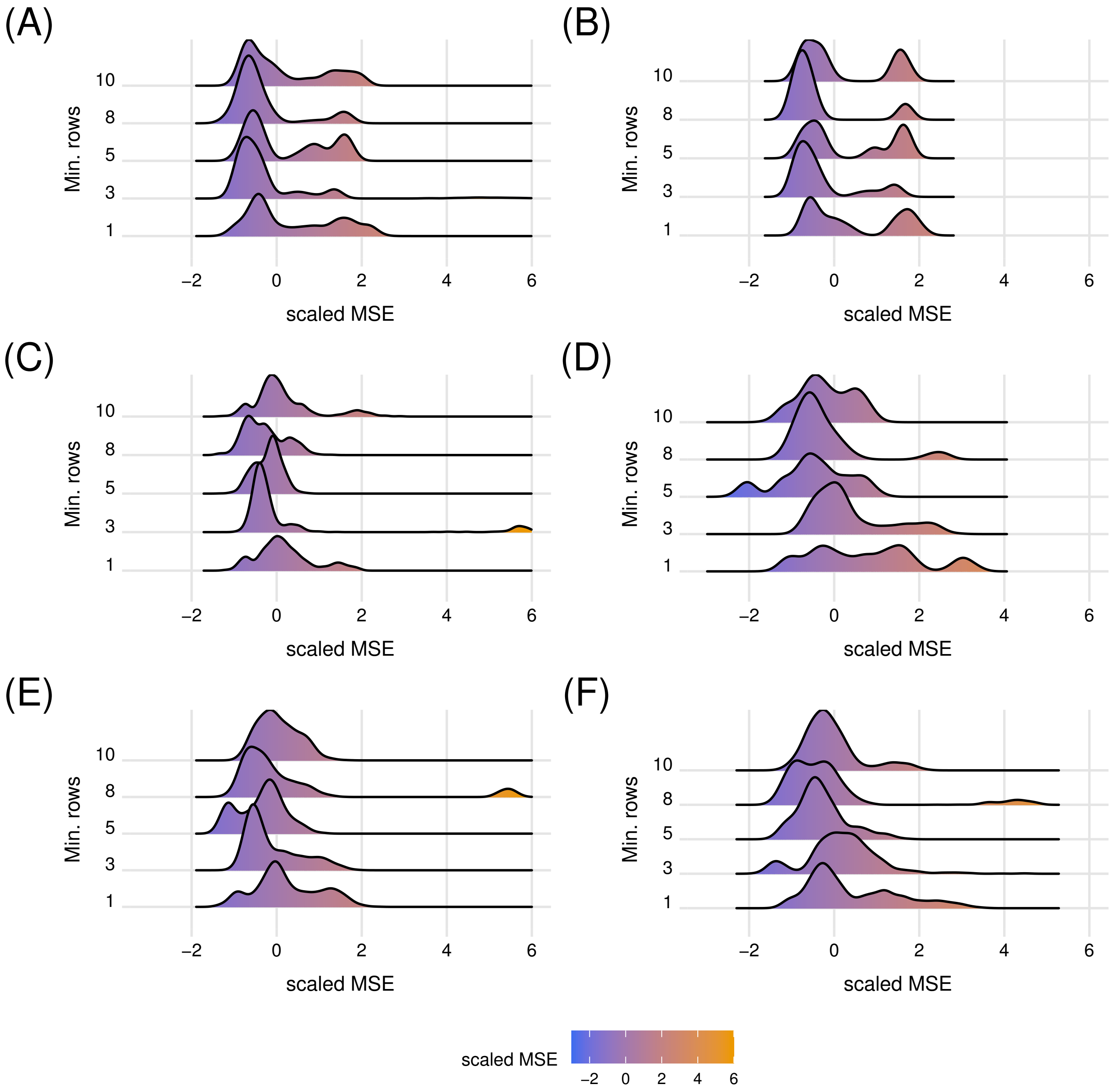

Figure S6. Distribution of the scaled mean squared error (MSE) per hyperparameter level of minimum number of rows required for further splitting. Error of every grid search model was scaled by subtracting the mean and dividing it by the standard deviation of the respective cross validation split. Datasets: **(A)**: Ra1, **(B)**: Ra2, **(C)**: Ra3, **(D)**: Wh1, **(E)**: Co1, **(F)**: Co2

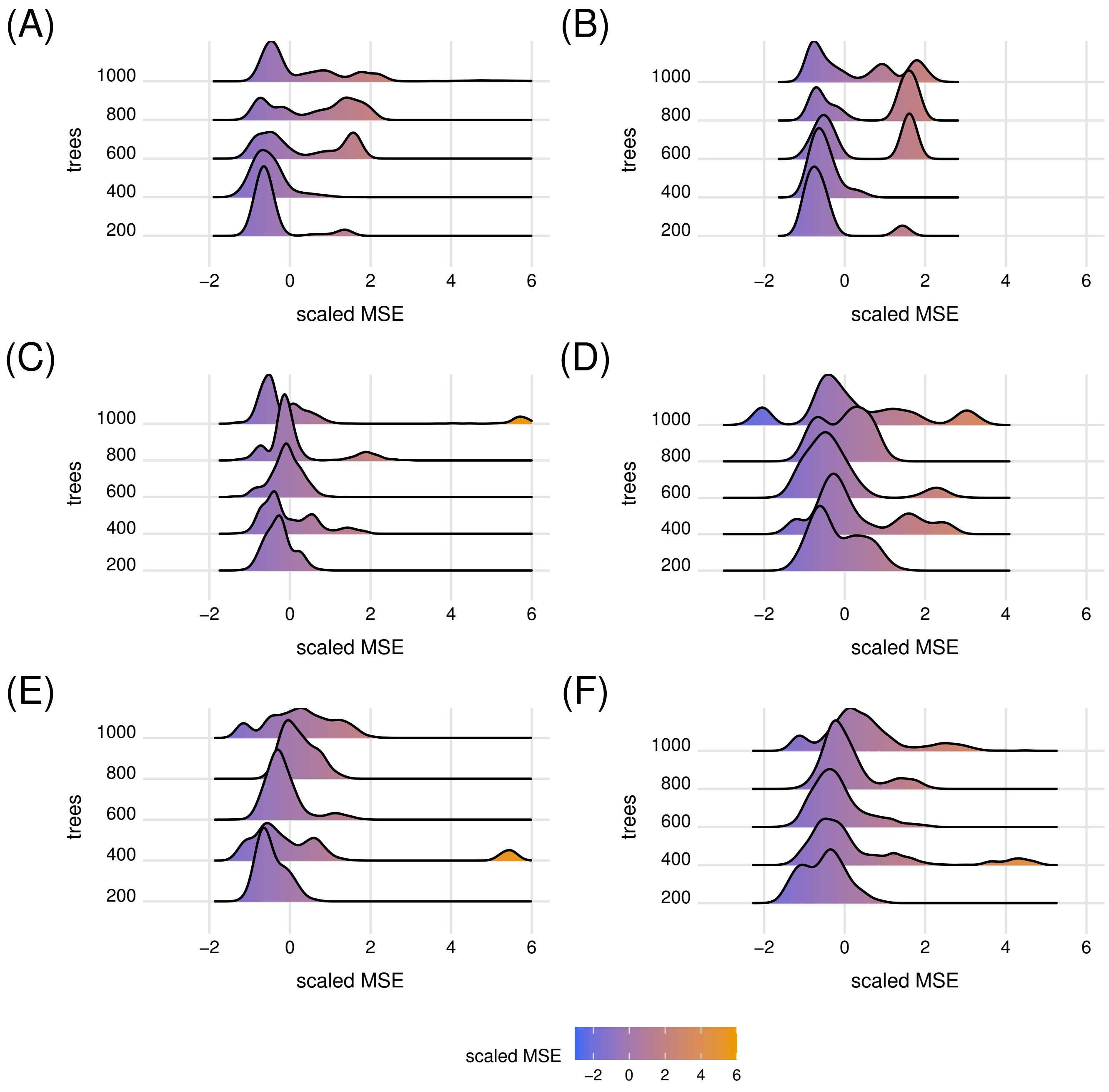

Figure S7. Distribution of the scaled mean squared error (MSE) per hyperparameter level of number of trees used to train a model. Error of every grid search model was scaled by subtracting the mean and dividing it by the standard deviation of the respective cross validation split. Datasets: **(A)**: Ra1, **(B)**: Ra2, **(C)**: Ra3, **(D)**: Wh1, **(E)**: Co1, **(F)**: Co2

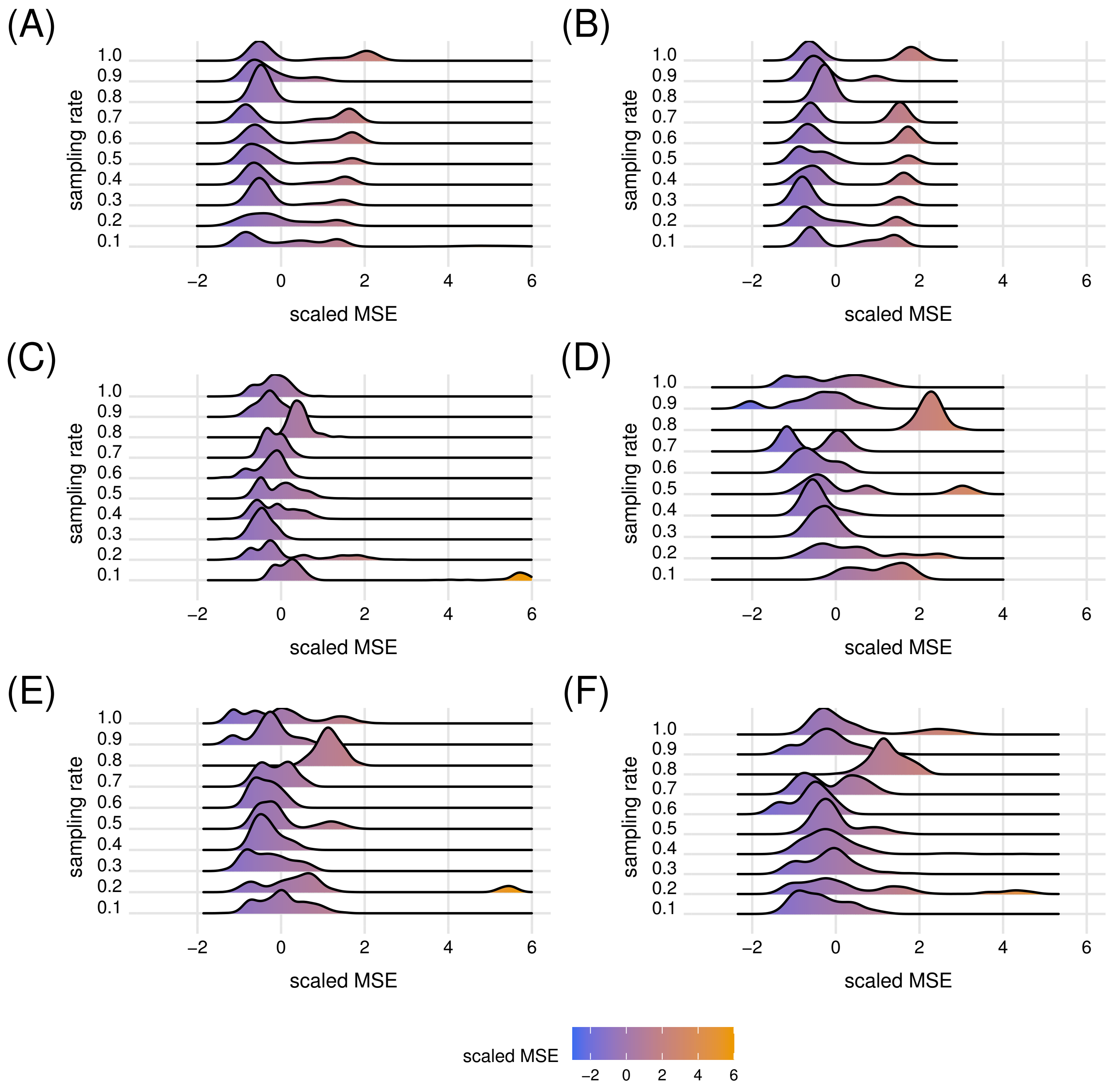

Figure S8. Distribution of the scaled mean squared error (MSE) per hyperparameter level of the row sampling rate. Error of every grid search model was scaled by subtracting the mean and dividing it by the standard deviation of the respective cross validation split. Datasets: **(A)**: Ra1, **(B)**: Ra2, **(C)**: Ra3, **(D)**: Wh1, **(E)**: Co1, **(F)**: Co2

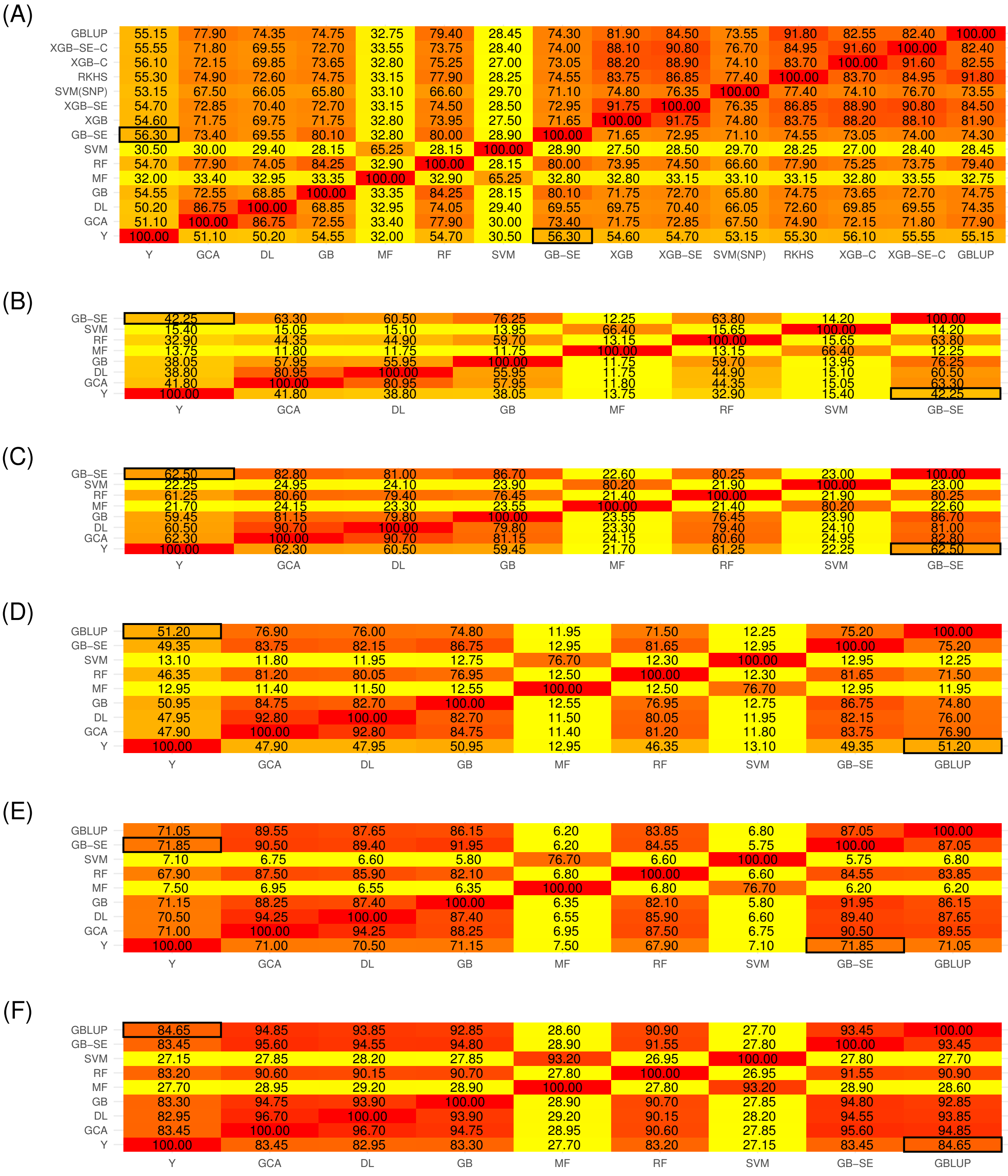

Figure S9. Heatmap indicating the overlap for the best 20 hybrids between two prediction methods. Numbers display the percentage of hybrids occuring in the top 20 of both methods according to predicted yield. 'Y' represents the true top 20 highest yielding hybrids. Black boxes indicating the highest overlap between a prediction method and the true top 20. Datasets: **(A)**: Ra1, **(B)**: Ra2, **(C)**: Ra3, **(D)**: Wh1, **(E)**: Co1, **(F)**: Co2

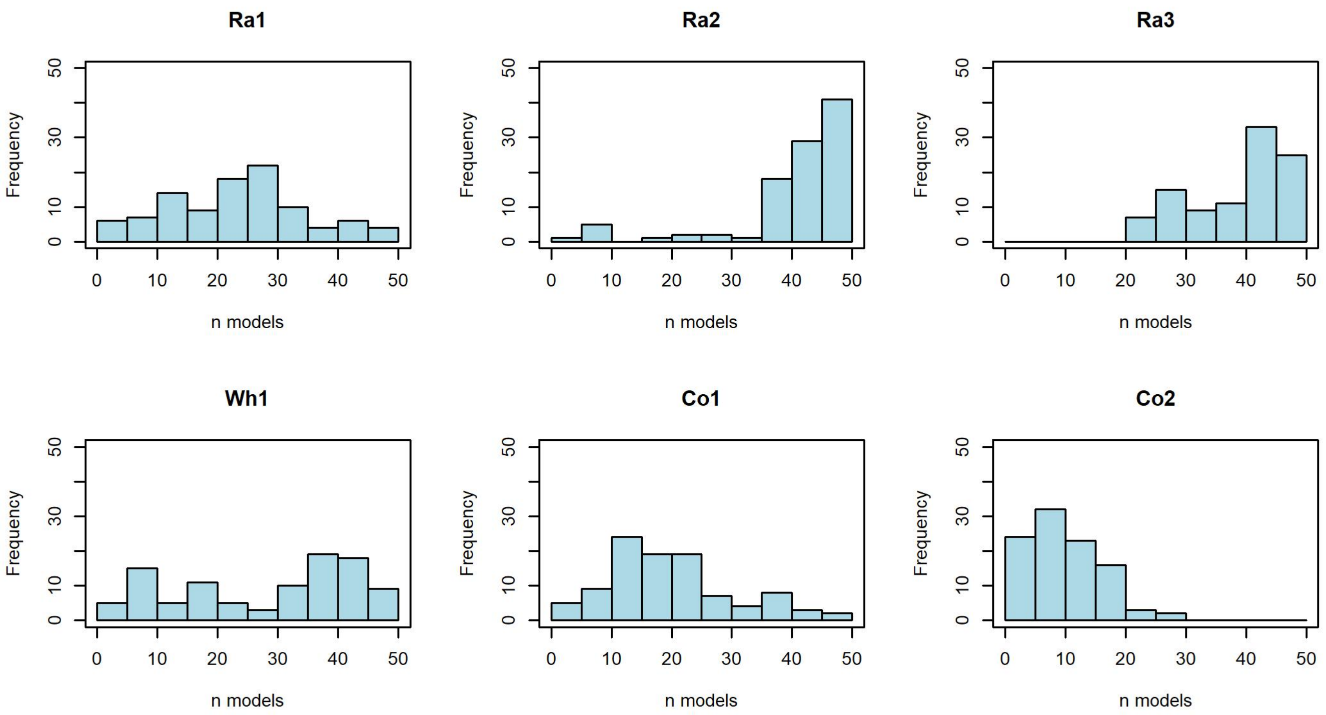

Figure S10. Optimum number of models to include in a GB-SE for each dataset. Every bar indicates the frequency of how often a certain number of models was considered the optimum number of models to be included in the final GB-SE.

1.2 Tables

Table S1. Overview of all possible hyperparameters considered in the grid search. Names of the listed hyperparameters correspond to the argument names used in h2o. A short description of each hyperparameter and their common names can be found in the materials and methods section of the paper.

Hyperparameter	Gridparameters
GBM & RF	
n_trees	$(200, 400, \ldots, 1000)$
max_depth	$(2, 4, \ldots, 20)$
min_rows	$(1, 3, 5, 8, 10)$
sample_rate	$(0.1, 0.2, \ldots, 1.0)$
nbins_cats	$(N_{pl} \cdot 0.05, N_{pl} \cdot 0.25, N_{pl} \cdot 0.5, N_{pl})$†
learn_rate ‡	0.1
XGB	
n_trees	(5000)
max_depth	$(2, 4, \ldots, 20)$
min_rows	$(1, 5, 10)$
sample_rate	$(0.2, 0.4, \ldots, 1.0)$
col_sample_rate_by_tree	$(0.2, 0.4, \ldots, 1.0)$
gamma	$(0, 5, 10, 20)$
learn_rate	$(0.01, 0.05, 0.1)$
nbins_cats	$(N_{pl} \cdot 0.05, N_{pl} \cdot 0.25, N_{pl} \cdot 0.5, N_{pl})$†
ANN	
hidden	$64, 128, (32, 32), (64, 32), (128, 64), (64, 64, 64)$
epochs	$(2, 4, 10, 20)$
input_dropout	$(0, 0.2)$
rate	$(0.001, 0.0005, 0.0001)$
SVM	
C	$(2^2, 2^3, \ldots, 2^9)$
epsilon	$(0.00, 0.01, \ldots, 0.20)$
kernel	linear, polynomial, radial
degree	$(2, 3, \ldots, 9)$
MF	
dim	$(4, 8, \ldots, 20)$
niter	500
costp_l1	$(0, 0.01)$
costq_l1	$(0, 0.01)$
lrate	0.05

† N_{pl} is the sum of all parental lines in the training set ‡ Only for GB

Table S2. GB hyperparameter combinations for each dataset with no. times best ≥ 5. A model was considered the best if it achieved the lowest error within a grid search. Only grid search runs with nominal parent information were considerd.

Dataset	Model No.	max_depth	min_rows	nbins_cats	ntrees	sample_rate	no. times best
Ra1	36	2	8	5%	400	0.2	80
	44	18	3	50%	600	0.7	6
	45	10	3	25%	600	0.5	5
Ra2	43	8	8	25%	200	0.2	26
	17	16	8	25%	200	0.4	24
	22	16	8	25%	200	0.3	23
	27	8	3	25%	400	0.5	10
	45	10	3.	25%	600	0.5	12
Ra3	5	4	8	50%	600	0.6	62
	46	14	10	50%	400	0.2	9
	19	10	8	50%	1000	0.3	8
	31	10	8	50%	800	0.2	8
	23	4	5	25%	400	1.0	6
Wh1	14	2	5	50%	1000	0.9	100
Co1	14	2	5	50%	1000	0.9	45
	23	4	5	25%	400	1.0	42
	9	4	1	25%	400	0.3	8
Co2	34	2	3	25%	200	0.6	71
	43	8	8	25%	200	0.2	11
	14	2	5	50%	1000	0.9	8

Table S3. Complete overview of the count of all best performing GB models for each dataset. Numbers indicate the times a model was considered the best, i.e. achieved the lowest mean square error within a grid search. Models without any case of being considered the best were removed from the table. Only grid search runs with nominal parent information were considered.

Model No.	Ra1	Ra2	Ra3	Wh1	Co1	Co2
5	0	0	62	0	0	0
7	0	0	3	0	0	0
9	0	0	0	0	8	0
10	1	0	0	0	0	0
11	0	0	0	0	0	1
14	0	0	0	100	45	8
15	0	1	0	0	0	0
16	1	0	0	0	1	3
17	1	24	0	0	1	4
19	0	1	8	0	0	0
20	0	1	0	0	0	0
22	0	23	0	0	0	2
23	0	0	6	0	42	0
24	0	0	4	0	0	0
26	1	0	0	0	0	0
27	1	10	0	0	0	0
31	0	0	8	0	0	0
34	1	2	0	0	0	71
36	80	0	0	0	0	0
42	1	0	0	0	0	0
43	0	26	0	0	3	11
44	6	0	0	0	0	0
45	5	12	0	0	0	0
46	0	0	9	0	0	0
48	1	0	0	0	0	0
50	1	0	0	0	0	0

1.3 Code Example

```
### Heilmann et al. 2023
# Example procedure to conduct a Gradient Boosting Machine grid search
# and form Stacked Ensembles on the basis of the results using h2o
# and a corn dataset from Technow et al. 2014

# Load required packages
library(sommer)
library(h2o)

# Start the h2o cluster
h2o.init()

# Function to restart after each iteration to prevent memory cluttering
restart <- function(nodes = 4){
  h2o.shutdown(F)
  h2o.init(nthreads = nodes)
  Sys.sleep(1)  # Sometimes needs some time to start up
}

# Load the dataset
data("DT_technow")

# Categorical variables are required to be of type 'factor'
DT_technow$dent   <- as.factor(DT_technow$dent)
DT_technow$flint  <- as.factor(DT_technow$flint)
DT_technow$hy     <- as.factor(DT_technow$hy)

### Hyperparameter search space for grid search
# All of these hyperparameter levels will be considered in the random selection
# of hyperparameter combinations used in the random grid search.
# Can be extended or modified

params <- list(ntrees      = seq(200,1000,200),     # Number of trees to include
               max_depth   = seq(2, 20, 2),         # Max. depth of each tree
               min_rows    = c(1,3,5,8,10),         # Min. rows required for split
               sample_rate = seq(0.1, 1.0, 0.1),    # Fraction of rows sampled per tree
               nbins_cats  = NA)                    # gets replaced later

search_criteria <- list(strategy   = "RandomDiscrete", # Set for random grid search
                        max_models = 10,               # Train 50 models
                        seed       = 2102)             # Seed for reproducibility

# Creates list 'idx.list' with indices for 100 random splits
# Split ratio was 90% training set to 10% test set
# Splits can be generated again by setting the seed and sending
# the command again. This is to ensure somewhat reproducible results.

seed.vec <- 2017
idx.list <- list()
set.seed(seed.vec)

# Each iteration creates a random split, stores it in a list
for (x in 1:100) {
  idx.list[[x]] <- sample(x    = 1:nrow(DT_technow),
                          size = round(nrow(DT_technow)*0.9))
}


# Seeds for the random processes within the ML algorithm
# Not to be confused with seed set for choosing
# the hyperparameters.
# Setting this seed does not provide 100% reproducibility
# but results are closer to each other
set.seed(123123)
lseed <- sample(1:10000, 100)
```

```r
gbm_cors  <- c()     # To store gbm prediction accuracy
ens_cors  <- c()     # To store ensemble prediction accuracy
grid_list <- list()  # To store grid details

for (i in 1:100) {
  # Get the indicies for the training set in this iteration
  idx   <- idx.list[[i]]

  # Assign training set by index
  Train <- DT_technow[idx, ]

  # Hence, removing the training set indices leaves the test set
  Test  <- DT_technow[-idx, ]

  # Check if any parental line are contained in the test set but not the
  # training set. If such is the case, remove them from the training set
  # Both parental lines need to be available in the training set for GCA
  # to work

  if (!all(Test$dent %in% Train$dent)) {
    Test <- Test[Test$dent %in% Train$dent, ]
  }
  if (!all(Test$flint %in% Train$flint)) {
    Test <- Test[Test$flint %in% Train$flint, ]
  }

  # Dataframes need to be transformed into h2o.frames for further use
  Train.h2o <- as.h2o(Train[c("GY", "flint", "dent")])
  Test.h2o  <- as.h2o( Test[c("GY", "flint", "dent")])

  # We set the nbins_cats hyperparameter space according
  # to the number of factor levels, i.e. parental lines, in our
  # training set. Depending on the train/test split, this may change
  n_cat <- length(levels(Train$flint)) + length(levels(Train$dent))

  # Modify the hyperparameter search space
  # We use 100%, 50%, 25% or 5% of factor levels
  params$nbins_cats <- round(c(n_cat, n_cat * .5, n_cat * .25, n_cat * .05))

  # Run the grid search
  # Depending on the computational capacity and threads used, this may take a while
  # Using the standard metric 'mean residual deviance' is identical to MSE for this task
  gbm_grid <-
    h2o.grid( algorithm       =  "gbm",                    # Algorithm for grid search
              y               = "GY",                      # Target variable
              x               = c("flint", "dent"),        # Predictor variables
              training_frame  = Train.h2o,                 # Data, must be in h2o format
              grid_id         = paste0("gbm_grid_", i),    # Name of the grid
              nfolds          = 10,                        # 10-fold CV
              seed            = lseed[i],                  # seed for random procedures
              hyper_params    = params,                    # predefined hyperparameters
              search_criteria = search_criteria,           # predefined criteria
              keep_cross_validation_predictions = TRUE     # save CV - required for SE
    )

  # Get best grid search model
  best_model <- h2o.getModel(gbm_grid@model_ids[[1]])

  # Make predictions with best model, transform h2o.frane to dataframe
  gbm_preds <- as.data.frame(h2o.predict(best_model, Test.h2o))[, ]

  # Store accuracy in vector
  gbm_cors  <- c(gbm_cors, cor(Test$GY, gbm_preds, method = "pearson"))
```

```
  # Visualize predicted compared to observed yield
  plot(
    Test$GY,
    gbm_preds,
    pch = 20,
    col = "black",
    xlim = c(min(Test$GY),   max(Test$GY)),
    ylim = c(min(gbm_preds), max(gbm_preds))
  )

  # Store grid details and ranking in a list
  grid_list[[i]] <- as.data.frame(gbm_grid@summary_table)


  ### This finds the optimum number of models for the
  ### Stacked Ensemble and predicts yield using the best

  # Create dataframe to store number of models
  # and prediction accuracy
  ensemble_eval <- data.frame()

  # Iterate over sequence of Top 5, 10, ..., 50 models
  for (x in seq(5,50,5)) {
    ensemble <-
      h2o.stackedEnsemble(
        y                      = "GY",                                # target
        x                      = c("flint", "dent"),                  # predictors
        metalearner_algorithm = "glm",                                # super learner
        metalearner_params    = list(lambda_search = T, alpha = 0),   # Ridge Regression
        metalearner_nfolds    = 10,                                   # 10-fold CV
        training_frame        = Train.h2o,                            # in h2o format
        base_models           = c(unlist(gbm_grid@model_ids[1:x]))    # model names
      )

    # Save the r2 as a measure of prediction accuracy here
    r2 <- ensemble@model$metalearner_model@model$cross_validation_metrics@metrics$r2
    r2 < as.vector(r2)

    # Store no. of models included and accuracy in dataframe
    ensemble_eval = rbind(ensemble_eval,c(x,r2) )
  }

  # Choose no. of models with highest accuracy
  ens_model_num <- ensemble_eval[which.max(ensemble_eval[,2]),1]
  # n_models <- c(n_models,ens_model_num)

  # Build Stacked Ensemble with best no. of models
  ensemble <-
    h2o.stackedEnsemble(
      y                      = "GY",
      x                      = c("flint", "dent"),
      metalearner_algorithm = "glm",
      metalearner_params    = list(lambda_search = T, alpha = 0),
      metalearner_nfolds    = 10,
      training_frame        = Train.h2o,
      base_models           = c(unlist(gbm_grid@model_ids[1:ens_model_num]))
    )

  # Make predictions with best ensemble, transform h2o.frane to dataframe
  ens_preds <- as.data.frame(h2o.predict(ensemble, Test.h2o))[, ]

  # Store accuracy in vector
  ens_cors <- c(ens_cors, cor(Test$GY, ens_preds, method = "pearson"))
}
```
